# Association of Radiation and Procarbazine Dose With Risk of Colorectal Cancer Among Survivors of Hodgkin Lymphoma

**DOI:** 10.1001/jamaoncol.2022.7153

**Published:** 2023-02-02

**Authors:** Yvonne M. Geurts, Rebecca Shakir, Georgios Ntentas, Sander Roberti, Marianne C. Aznar, Katinka M. John, Johanna Ramroth, Cécile P. M. Janus, Augustinus D. G. Krol, Judith M. Roesink, Richard W. M. van der Maazen, Josée M. Zijlstra, Sarah C. Darby, Berthe M. P. Aleman, Flora E. van Leeuwen, David J. Cutter, Michael Schaapveld

**Affiliations:** 1 Department of Epidemiology and Biostatistics, the Netherlands Cancer Institute, Amsterdam, the Netherlands; 2 Nuffield Department of Population Health, University of Oxford, Oxford, UK; 3 Department of Medical Physics, Guy’s and St Thomas’ NHS Foundation Trust, London, UK; 4 School of Biomedical Engineering and Imaging Sciences, King’s College London, London, UK; 5 Division of Cancer Sciences, Faculty of Biology, Medicine, and Health, University of Manchester, Manchester, UK; 6 Department of Radiotherapy, Erasmus Medical Center, Rotterdam, the Netherlands; 7 Department of Radiation Oncology, Leiden University Medical Center, Leiden, the Netherlands; 8 Department of Radiotherapy, University Medical Center Utrecht, Utrecht, the Netherlands; 9 Department of Radiation Oncology, Radboud University Medical Center, Nijmegen, the Netherlands; 10 Department of Hematology, Amsterdam University Medical Center, Vrije Universiteit, Cancer Center Amsterdam, Amsterdam, the Netherlands; 11 Department of Radiation Oncology, the Netherlands Cancer Institute, Amsterdam, the Netherlands; 12 Oxford Cancer and Hematology Center, Oxford University Hospitals National Health Service Foundation Trust, Churchill Hospital, Oxford, UK

## Abstract

**Importance:**

Hodgkin lymphoma (HL) survivors have higher rates of colorectal cancer, which may be associated with subdiaphragmatic radiation therapy and/or alkylating chemotherapy. Although radiation dose-response associations with breast, lung, stomach, pancreatic, and esophageal cancer after HL have been demonstrated, the association of radiation therapy with colorectal cancer remains unclear.

**Objective:**

To quantify the rate of colorectal cancer according to radiation dose to the large bowel and procarbazine dose among HL survivors.

**Design, Setting, and Participants:**

A nested case-control study examined 5-year HL survivors at 5 hospital centers in the Netherlands. Participants had been diagnosed with HL in 1964 to 2000, when they were 15 to 50 years of age, and were followed for a median of approximately 26 years. Survivors of HL who developed colorectal cancer and survivors who were selected as controls were individually matched on sex, age at HL diagnosis, and date of HL diagnosis. Data were analyzed from July 2021 to October 2022.

**Exposures:**

Mean radiation doses to the large bowel were estimated by reconstructing individual radiation therapy treatments on representative computed tomography data sets.

**Main Outcomes and Measures:**

Excess rate ratios (ERRs) were modeled to evaluate the excess risk associated with each 1-gray increase in radiation dose, and potential effect modification by procarbazine was explored.

**Results:**

The study population included 316 participants (mean [SD] age at HL diagnosis, 33.0 [9.8] years; 221 [69.9%] men), 78 of whom were HL survivors who developed colorectal cancer (cases) and 238 who did not (controls). The median (IQR) interval between HL and colorectal cancer was 25.7 (18.2-31.6) years. Increased colorectal cancer rates were seen for patients who received subdiaphragmatic radiation therapy (rate ratio [RR], 2.4; 95% CI, 1.4-4.1) and those who received more than 8.4 g/m^2^ procarbazine (RR, 2.5; 95% CI, 1.3-5.0). Overall, colorectal cancer rate increased linearly with mean radiation dose to the whole large bowel and dose to the affected bowel segment. The association between radiation dose and colorectal cancer rate became stronger with increasing procarbazine dose: the ERR per gray to the whole bowel was 3.5% (95% CI, 0.4%-12.6%) for patients who did not receive procarbazine, and increased 1.2-fold (95% CI, 1.1-1.3) for each 1-g/m^2^ increase in procarbazine dose.

**Conclusions and Relevance:**

This nested case-control study of 5-year HL survivors found a dose-response association between radiation therapy and colorectal cancer risk, and modification of this association by procarbazine. These findings may enable individualized colorectal cancer risk estimations, identification of high-risk survivors for subsequent screening, and optimization of treatment strategies.

## Key Points

**Question:**

Is there a dose-response association of radiation dose to the large bowel and/or procarbazine dose with subsequent colorectal cancer risk in Hodgkin lymphoma survivors?

**Findings:**

This nested case-control study of 316 patients who underwent treatment for Hodgkin lymphoma at 5 hospitals in the Netherlands found a linear dose-response association between radiation dose to the large bowel and colorectal cancer risk; the dose-response association became steeper with higher doses of procarbazine.

**Meaning:**

The findings of this case-control study support the use of colorectal cancer screening for survivors of Hodgkin lymphoma who have been treated with subdiaphragmatic radiation therapy and/or procarbazine; this dose-response association can be used to estimate the risk of colorectal cancer among these patients.

## Teaser

This nested case-control study of 5-year Hodgkin lymphoma survivors explores dose-response associations of radiation therapy and procarbazine with colorectal cancer.

## Introduction

Advances in Hodgkin lymphoma (HL) treatment have yielded improvements in survival; 5-year disease-specific survival is now greater than 85%.^1^ However, survivors are at risk of treatment-related late adverse effects, including subsequent malignant neoplasms.^2,3,4,5,6,7,8,9,10,11,12^ We have previously shown that gastrointestinal malignant tumors are the third most common second cancers in HL survivors, after breast and lung cancer.^12^ Compared with the general population, HL survivors have a 2.8-fold higher rate of colorectal cancer, and the rate remains increased for more than 40 years.^12^ We also previously reported on associations of subdiaphragmatic RT and procarbazine with increased colorectal cancer rates among HL survivors.^10^ However, those results cannot be used to estimate colorectal cancer rates for patients treated more recently because radiation therapy (RT) and chemotherapy doses are now lower. Therefore, an understanding of the dose-response relationship is needed to quantify and estimate the risk of secondary cancers based on individual treatment exposures. These relationships have been demonstrated for secondary cancers of the breast,^13,14^ lung,^15^ stomach,^16^ pancreas,^17^ and esophagus^18^ after treatment for HL, but not for the colon or rectum, to our knowledge. To quantify the colorectal cancer rate associated with radiation dose to the large bowel and cumulative procarbazine dose, we have conducted a nested case-control study using individual HL treatment data.

## Methods

This study was reviewed and deemed to be exempt from institutional review board approval and informed consent per Dutch law regarding studies that use only existing data from medical files. The study followed the Strengthening the Reporting of Observational Studies in Epidemiology (STROBE) reporting guideline.

### Study Population

Participants were selected from a multicenter cohort of 2996 patients who had survived HL for 5 years or longer and who had been diagnosed in 1964 to 2000; they were 15 to 50 years of age at first treatment.^12^ All patients had been treated with RT (supradiaphragmatic and/or subdiaphragmatic) and/or chemotherapy. In total, 83 patients who had potential colorectal cancer were identified; 5 were excluded per eligibility criteria (eTable 1 in Supplement 1). For each case (patient with colorectal cancer), up to 5 participants were selected to be a control; they were individually matched on sex, age at HL diagnosis (≤3 years), and date of HL diagnosis (≤4 years; details are available in the eMethods and eTables 2 and 3 in Supplement 1). The control participants were selected by incidence density sampling, and all survivors remained eligible to be in the control group until they experienced a case-defining event or were censored.^19^ Survivors could serve as a control for more than 1 case in the colorectal cancer group (eMethods in Supplement 1).

### Data Collection

Details of the HL treatments used during the study period are presented in the eMethods in Supplement 1. Data on patient characteristics, including height and weight, HL stage, and treatments for primary and relapsed disease (as applicable) were extracted from medical files. Chemotherapy details collected included dates of administration, regimens, number of cycles, drugs, dosages, and indication (primary or relapse). Details of RT included the original radiation prescription records, planning calculations, and simulation films (eFigure 1 in Supplement 1). Where original prescription records were unavailable, data on RT, including dates, field type, dose, fractionation, and treatment energy, were collected. For cases of colorectal cancer, additional information on the colorectal cancer diagnosis was recorded, including the topography as per the *International Classification of Diseases for Oncology*.^20^

### Radiation Dosimetry

Twelve computed tomography (CT) data sets (6 male and 6 female) were chosen from a library of 66 patients with HL to be representative of anatomy. The large bowel was contoured according to published guidelines,^21,22^ and anatomical landmarks were used to define segments.^23^ Supra- and subdiaphragmatic treatment fields, shielding, and prescribed doses were reproduced from original RT prescriptions and simulation films (eFigure 1 in Supplement 1). Mean doses were estimated for the whole large bowel and for all segments. If no information was available on prescribed radiation dose, energy, or field borders, it was imputed from other individuals treated at the same period and center with the same field types (eMethods in Supplement 1).

### Statistical Analysis

Cumulative procarbazine doses were estimated using typical doses (grams per meter squared [g/m^2^]) per cycle for each chemotherapy regimen. The colorectal cancer odds ratios (ORs) and CIs were calculated using conditional logistic regression,^24^ comparing the exposure history of cases with those of matched controls. The resulting ORs can be interpreted as rate ratios (henceforth referred to as RR).^25^ Patients with unknown RT dose were excluded from tests for heterogeneity and trend. All models were fitted using maximum likelihood.

Excess rate ratios (ERRs) were estimated to evaluate the excess risk associated with each gray (Gy) increase in radiation dose (ERR/Gy). The overall ERR/Gy (ie, without effect modification) was estimated using a linear dose-response model:

RR = K_m_(1 + βd_rad_)

where d_rad_ was the radiation dose to the whole large bowel (d_rad whole_) or the affected segment (d_rad seg_), K_m_ was the constant specific to each matched set, and β was the proportional increase in colorectal cancer rate per 1-Gy increase in radiation dose. Nonlinearity was evaluated by including a curvature term:
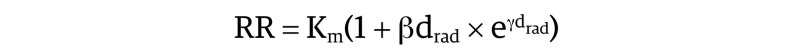
and departure from linearity was evaluated by a likelihood ratio test of the hypothesis γ = 0. Confidence intervals for linear model parameters were based on the profile likelihood. Controls for whom radiation dose was unknown (n = 1) were excluded from any model including radiation dose. If radiation dose was unknown for a case (n = 1), the full case-control set was excluded from analysis.

Heterogeneity in ERRs for radiation dose according to patient and treatment characteristics (sex, age at HL diagnosis, follow-up interval, alkylating chemotherapy, anthracyclines, and procarbazine dose) was evaluated by including an interaction term with the characteristic as a categorized variable:

RR = K_m_(1 + β_category of characteristic_ × d_rad_)

and a likelihood ratio test. Modification of the radiation dose response by procarbazine dose was explored using the following 2 models:

RR = K_m_(1 + βd_rad_ + θd_rad_ × d_proc_)

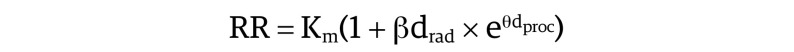
in which cumulative procarbazine dose (d_proc_) was included as a continuous variable. The best fitting model was chosen based on the Akaike information criterion (AIC).^26^ A multivariable conditional logistic regression model with both radiation dose and procarbazine dose in categories was fitted. Linear trends within this model were tested by including 1 dose-variable as continuous and retaining the other as categorical.

We performed sensitivity analyses by excluding participants (1) who had received a cumulative procarbazine dose of ≥19.8 g/m^2^ (mean + 2 SDs); or (2) with imputed radiation doses for subdiaphragmatic fields (3 cases and 3 controls; eMethods in Supplement 1). To assess overestimation of the ERRs, sensitivity analyses using Firth correction were performed; there was no strong indication of overestimation.^27^

All statistical tests were 2-sided and *P* values of < .05 were considered statistically significant. Analyses were performed using Stata, version 15.1 (StataCorp LLC), Epicure, version 2.00.03 (Risk Sciences International),^28^ and R, version 4.1.1 (R Foundation for Statistical Computing), from July 2021 to October 2022.

## Results

### Patient Characteristics

The study population of 316 participants (mean [SD] age at HL diagnosis, 33.0 [9.8] years; 221 [69.9%] men; 95 [30.1%] women) included 78 patients with colorectal cancer (ie, cases; Table 1) and 238 control patients. The median (IQR) interval between HL and colorectal cancer diagnosis was 25.7 (18.2-31.6) years; and the median (IQR) age at colorectal cancer diagnosis was 59.1 (51.9-63.3) years. Fifty-six (72%) cases had colon cancer and 22 (28%) had rectal cancer. Fifty-four (69%) cases died (21 of colorectal cancer), and the colorectal cancer−specific survival at 5 years was 66%. Treatment for HL, including relapse, consisted of chemotherapy only (5% cases; 14% controls), RT only (26% cases; 37% controls), or combined modality therapy (69% cases; 50% controls; eTable 4 in Supplement 1).

**Table 1.** Characteristics of 78 Hodgkin Lymphoma (HL) Survivors Who Developed Colorectal Cancer (CRC) After Treatment

Characteristic	Cases, No. (%)^a^
Female sex	24 (31)
Male sex	54 (69)
Age at HL diagnosis, y	
15-24	21 (27)
25-34	24 (31)
35-50	33 (42)
Time of HL diagnosis	
1964-1976	35 (45)
1977-1988	31 (40)
1989-2000	12 (15)
Interval from HL to CRC, y	
5-14	15 (19)
15-24	23 (30)
25-34	29 (37)
≥35	11 (14)
Age at CRC diagnosis, y	
30-49	14 (18)
50-64	49 (63)
65-80	15 (19)
Stage of CRC at diagnosis	
I	9 (12)
II	14 (18)
III	12 (15)
IV	18 (23)
Unknown	25 (32)
**Site of CRC^b^**	
Colon	
Ascending	18 (23)
Transverse	12 (15)
Descending	24 (31)
NOS	2 (3)
Rectum	22 (28)
**Histologic findings**	
Mucinous	12 (15)
Adenocarcinoma, NOS	47 (60)
Unknown	19 (24)
**Status at completion of follow-up**	
Living	24 (31)
Deceased	54 (69)
CRC-related cause of death	21 (27)
Other cause of death	33 (42)

Abbreviation: NOS, not otherwise specified.

^a^ Percentages may not total 100 because of rounding.

^b^ CRCs were categorized as ascending (including cecum; n = 12) and right colon NOS (n=1); transverse (including hepatic flexure; n = 1) and splenic flexure (n = 0); descending, including sigmoid (n = 15); and rectosigmoid junction (n = 3) and rectum.

### Treatment-Related Risk Factors

**Figure 1.**
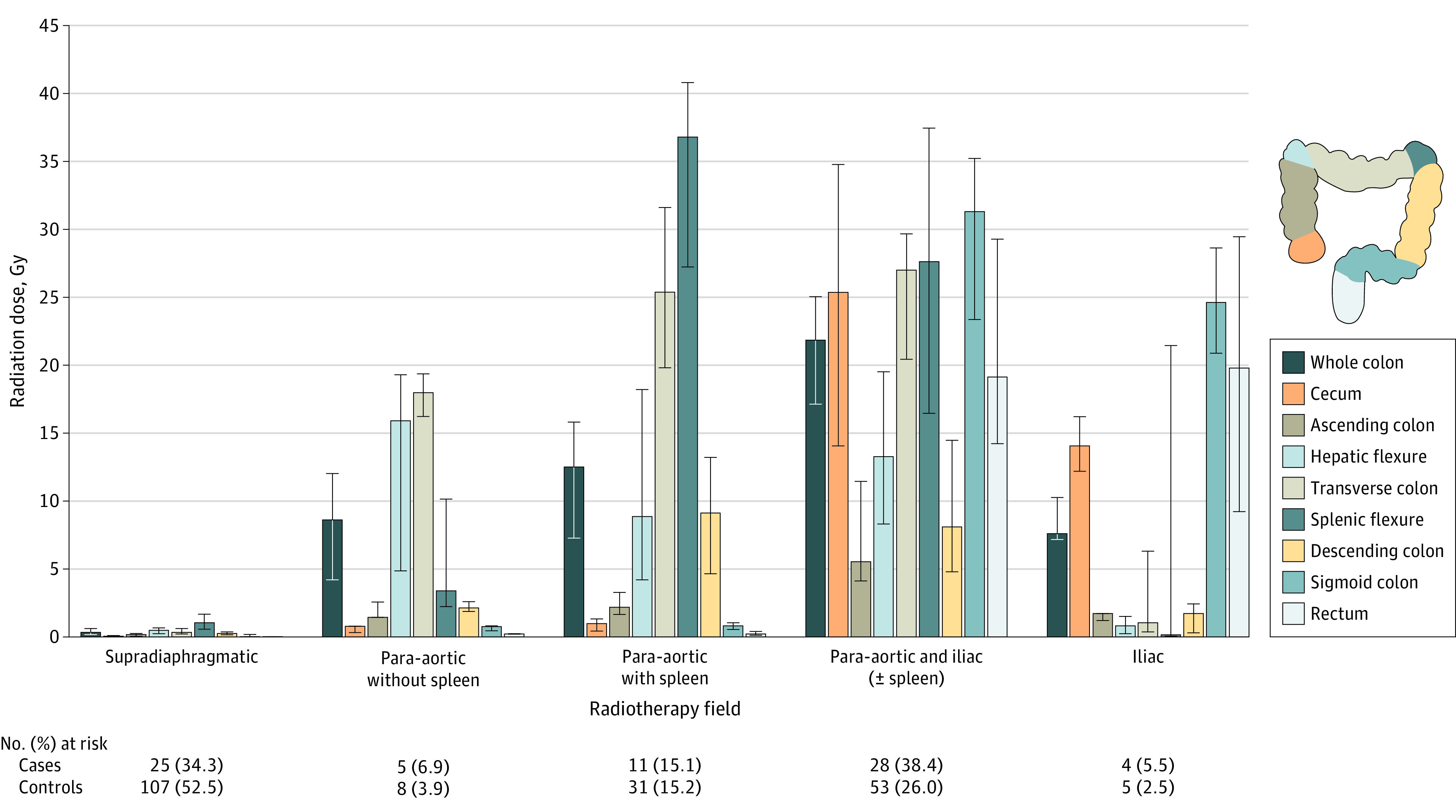
Median Mean Radiation Dose to the Large Bowel From Specific Radiation Therapy Fields Used for Hodgkin Lymphoma Treatment Radiation dose to large bowel segments for 73 cases and 204 controls (dose could not be reconstructed for 1 case and 1 control, both in the supradiaphragmatic radiotherapy category). Bars represent median mean radiation doses, and error bars represent IQRs. The position of the large bowel segments in reference to reconstructed fields from each of these categories is presented in eFigures 2 and 3 in Supplement 1.

In patients treated with RT, the median (IQR) mean radiation dose to the whole large bowel was 8.8 (0.6-20.2) Gy in cases and 1.6 (0.3-15.6) Gy in controls. Cases received subdiaphragmatic RT or relapse treatment more frequently than controls (subdiaphragmatic RT, 62% vs 41%; RR, 2.4; 95% CI, 1.4-4.1; relapse treatment, 37% vs 23%; RR, 2.1; 95% CI, 1.2-3.8, Table 2). The colorectal cancer rates were significantly increased (2.1 to 3.0-fold) for patients who received 10 or more Gy to the whole large bowel compared with patients who received no RT, or less than 1.0 Gy (ie, scattered radiation from supradiaphragmatic fields). The highest median mean dose to the whole large bowel (≥20 Gy) was delivered by the combination of para-aortic and iliac fields (with or without spleen; Figure 1). Para-aortic fields including the spleen were associated with high doses to the transverse colon and splenic flexure (median mean dose, ≥25 Gy); iliac fields were associated with high doses to the sigmoid colon and rectum (median mean dose, ≥19 Gy). eTable 5 in Supplement 1 shows the distribution of patient and treatment characteristics by radiation field.

**Table 2.** Univariable Modeled Treatment-Related Colorectal Cancer (CRC) Rates After Hodgkin Lymphoma (HL) Treatment^a^

Treatment factor	No. (%)		Rate ratio (95% CI)^b^	*P* value for heterogeneity	*P *value for trend^c^
	Cases (n = 78)	Controls (n = 238)			
**Treatment modality**					
Supradiaphragmatic RT only	4 (5.1)	37 (15.6)	1 [Reference]	<.001	NA
CT only or with supradiaphragmatic RT	26 (33.3)	104 (43.7)	2.6 (0.8-8.1)		
Subdiaphragmatic RT only	16 (20.5)	50 (21.0)	3.5 (1.0-11.5)		
CT and subdiaphragmatic RT	32 (41.0)	47 (19.8)	7.4 (2.3-23.7)		
**HL relapse treatment** ^d^					
No	49 (62.8)	184 (77.3)	1 [Reference]	.01	NA
Yes	29 (37.2)	54 (22.7)	2.1 (1.2-3.8)		
**RT field**					
No subdiaphragmatic	30 (38.5)	141 (59.2)	1 [Reference]	.001	NA
Any subdiaphragmatic fields	48 (61.5)	97 (40.8)	2.4 (1.4-4.1)		
**RT dose (median) to whole large bowel, Gy**					
No RT to <1.0 (0.22)	28 (35.9)	131 (55.0)	1 [Reference]	.01	<.001
1.0-9.9 (6.3)	13 (16.7)	31 (13.0)	1.6 (0.8-3.5)		
10.0-19.9 (15.3)	17 (21.8)	45 (18.9)	2.1 (1.0-4.5)		
≥20.0 (25.0)	19 (24.4)	30 (12.6)	3.0 (1.5-6.2)		
Unknown^e^	1 (1.3)	1 (0.4)	NA		
**RT dose (median) to affected segment, Gy** ^f^					
No RT to <1.0 (0.01)	38 (48.7)	161 (67.7)	1 [Reference]	.01	<.001
1.0-9.9 (3.4)	7 (9.0)	21 (8.8)	1.6 (0.6-4.3)		
10.0-19.9 (14.1)	8 (10.3)	16 (6.7)	2.3 (0.9-6.1)		
20.0-29.9 (24.8)	12 (15.4)	21 (8.8)	3.1 (1.3-7.4)		
≥30.0 (35.4)	12 (15.4)	18 (7.6)	3.3 (1.4-7.7)		
Unknown^e^	1 (1.3)	1 (0.4)	NA		
**Any chemotherapy**					
No	20 (25.6)	87 (36.6)	1 [Reference]	.09	NA
Yes	58 (74.4)	151 (63.4)	1.6 (0.9-2.9)		
**Procarbazine dose (median), g/m^2^** ^g^					
No procarbazine	27 (34.6)	113 (47.5)	1 [Reference]	.05	.003
1.0-4.2 (4.2)	9 (11.5)	35 (14.7)	0.9 (0.4-2.4)		
4.3-8.4 (8.4)	21 (26.9)	56 (23.5)	1.5 (0.8-3.0)		
>8.4 (14.0)	21 (26.9)	34 (14.3)	2.5 (1.3-5.0)		
**RT dose to whole large bowel and procarbazine dose, Gy and g/m^2^** ^g^ ^,^ ^h^ ^,^ ^i^					
<10 and ≤4.2	20 (26.0)	97 (40.9)	1 [Reference]	.002	NA
<10 and >4.2	21 (27.3)	65 (27.4)	1.8 (0.8-3.7)		
≥10 and ≤4.2	16 (20.8)	50 (21.1)	2.1 (1.0-4.8)		
≥10 and >4.2	20 (26.0)	25 (10.5)	5.2 (2.2-12.3)		

Abbreviations: ABV, doxorubicin, bleomycin, vinblastine; CRC, colorectal cancer; CT, chemotherapy; g/m^2^, grams per meter squared; Gy, gray; MOPP, combined mechlorethamine, vincristine, procarbazine, and prednisone; RT, radiation therapy.

^a^ Includes primary and relapse treatment.

^b^ Rate ratios for development of CRC were calculated conditionally on matched sets. Matching variables were sex, age at HL diagnosis, and date of HL diagnosis.

^c^ Test for linear trend with dose included as a continuous variable.

^d^ In total, 6 patients (1 case and 5 controls) underwent autologous stem cell transplantation for Hodgkin lymphoma relapse; none underwent allogeneic stem cell transplantation.

^e^ Patients with unknown RT dose were excluded from calculation *P* value for heterogeneity and *P* value for trend. If RT dose for a patient with CRC was unknown, the full case-control set was excluded from the analysis.

^f^ Mean RT dose to the affected large bowel segment (matched segment for controls).

^g^ Assuming a procarbazine dose of 1.4 g/m^2^ per cycle (14 days × 0.1 g/m^2^ per day), 4.2 g/m^2^ corresponds to 3 cycles and 8.4 g/m^2^ to 6 cycles of MOPP. Other protocols (eg, MOPP/ABV) include a procarbazine dose of 0.7 g/m^2^ per cycle.

^h^ Patients with unknown RT dose (1 case, 1 control) were not included. If RT dose for a patient with CRC was unknown, the full case-control set was excluded from the regression analysis.

^i^ There was no statistically significant additive (*P* = .15) or multiplicative (*P* = .56) interaction between RT dose to the whole large bowel in categories and procarbazine dose in categories. *P* values were calculated using a likelihood ratio test.

The median (IQR) cumulative procarbazine dose in patients who received procarbazine was 8.4 (6.0-13.0) g/m^2^ in cases compared with 8.0 (4.2-9.8) g/m^2^ in controls. Patients who received a cumulative procarbazine dose of more than 8.4 g/m^2^ had a significantly increased colorectal cancer rate (RR, 2.5; 95% CI, 1.3-5.0; Table 2) compared with patients who received no procarbazine. Patients who received both 10 Gy or more radiation to the whole large bowel and more than 4.2 g/m^2^ procarbazine had a 5.2-fold (95% CI, 2.2-12.3) increased colorectal cancer rate, compared with patients who received less than 10 Gy and 4.2 or less g/m^2^ procarbazine. Treatment with anthracyclines (RR, 1.0; 95% CI, 0.5-2.0), vinca-alkaloids (RR, 1.6; 95% CI, 0.9-2.9), any alkylating chemotherapy (RR, 1.6; 95% CI, 0.9-2.9), and splenectomy (RR, 1.1; 95% CI, 0.6-2.0) was not associated with increased colorectal cancer rate; nor was smoking (RR, 1.2; 95% CI, 0.7-2.1) or being overweight (RR, 1.0; 95% CI, 0.5-2.1; eTable 4 in Supplement 1) at HL diagnosis.

In multivariable conditional logistic regression with radiation and procarbazine dose in categories, we observed elevated RRs for radiation dose of 10 or more Gy to the whole large bowel (10.0-19.9 Gy [RR, 2.6; 95% CI, 1.2-5.6]; and ≥20.0 Gy [RR, 3.2; 95% CI, 1.5-6.6]) and cumulative procarbazine dose more than 8.4 g/m^2^(RR, 2.9; 95% CI, 1.4-6.1; eTable 6 in Supplement 1).

### Radiation Dose-Response Association

**Figure 2.**
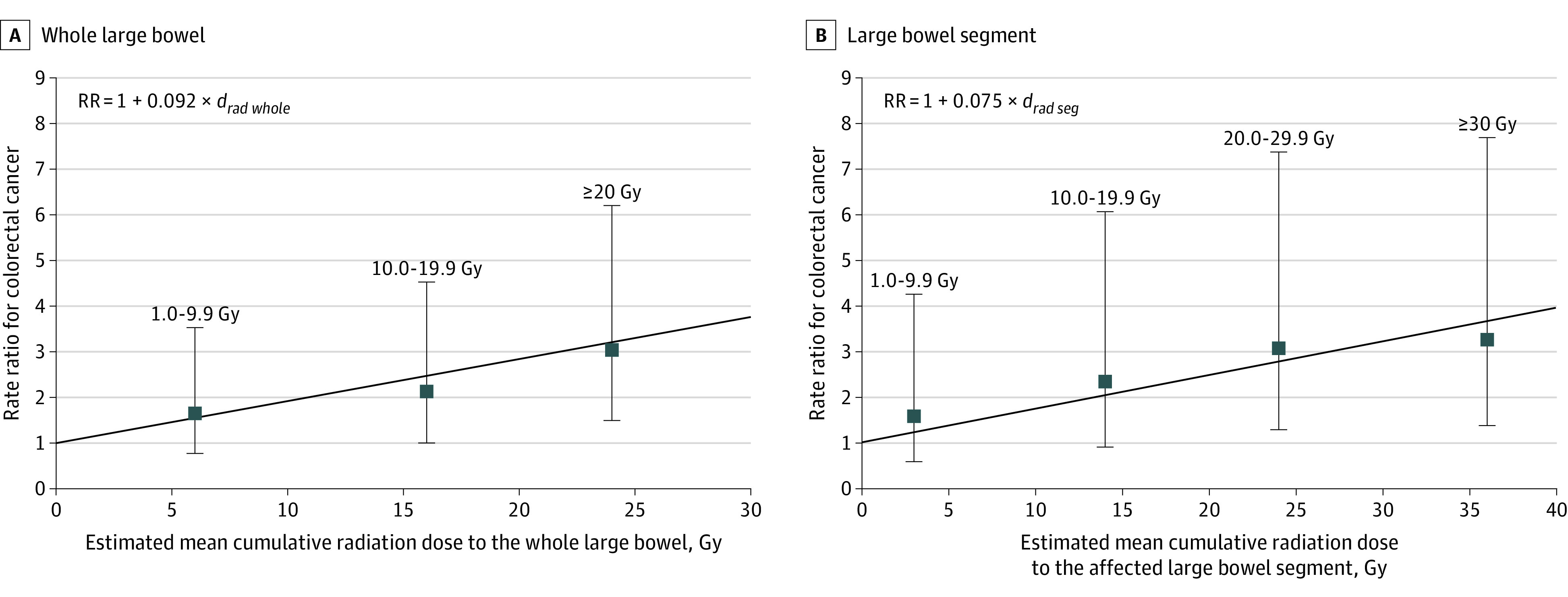
The Modeled Overall Radiation Dose-Response Association With Colorectal Cancer After Hodgkin Lymphoma Treatment These models represent overall dose-response association for radiation dose to the whole large bowel (A) and radiation dose to the affected large bowel segment (B) for patients included in this case-control study. The full models, considering the effect of both radiation dose and procarbazine dose are presented in Figure 3. Filled squares and error bars indicate rate ratios and 95% CIs for radiotherapy dose categories, plotted at the median dose of each category (reference category with median dose [0.2 Gy for dose to the whole large bowel and 0.01 Gy for dose to the affected segment] are shown in Table 2). Note that the radiation dose range differs for dose to the whole colon (up to 30 Gy) and dose to the affected segment (up to 40 Gy). The abbreviation d_rad seg_ refers to radiation dose to the affected large bowel segment; d_rad whole_, radiation dose to the whole large bowel; Gy, gray; and RR, rate ratio.

Overall, accounting for the matching factors but not the potential effect modifiers, colorectal cancer rate increased linearly with increasing mean radiation dose to the whole large bowel (overall ERR/Gy, 9.2%; 95% CI, 2.5%-23.0%; Figure 2A) and with mean dose to the affected segment (overall ERR/Gy, 7.5%; 95% CI, 2.2%-17.8%, Figure 2B). There was no significant curvature. When analysis was limited to cases with only colon cancer (not rectal cancer), the ERR/Gy colon dose was 9.0% (95% CI, 1.6%-26.6%; eFigure 4 in Supplement 1).

**Figure 3.**
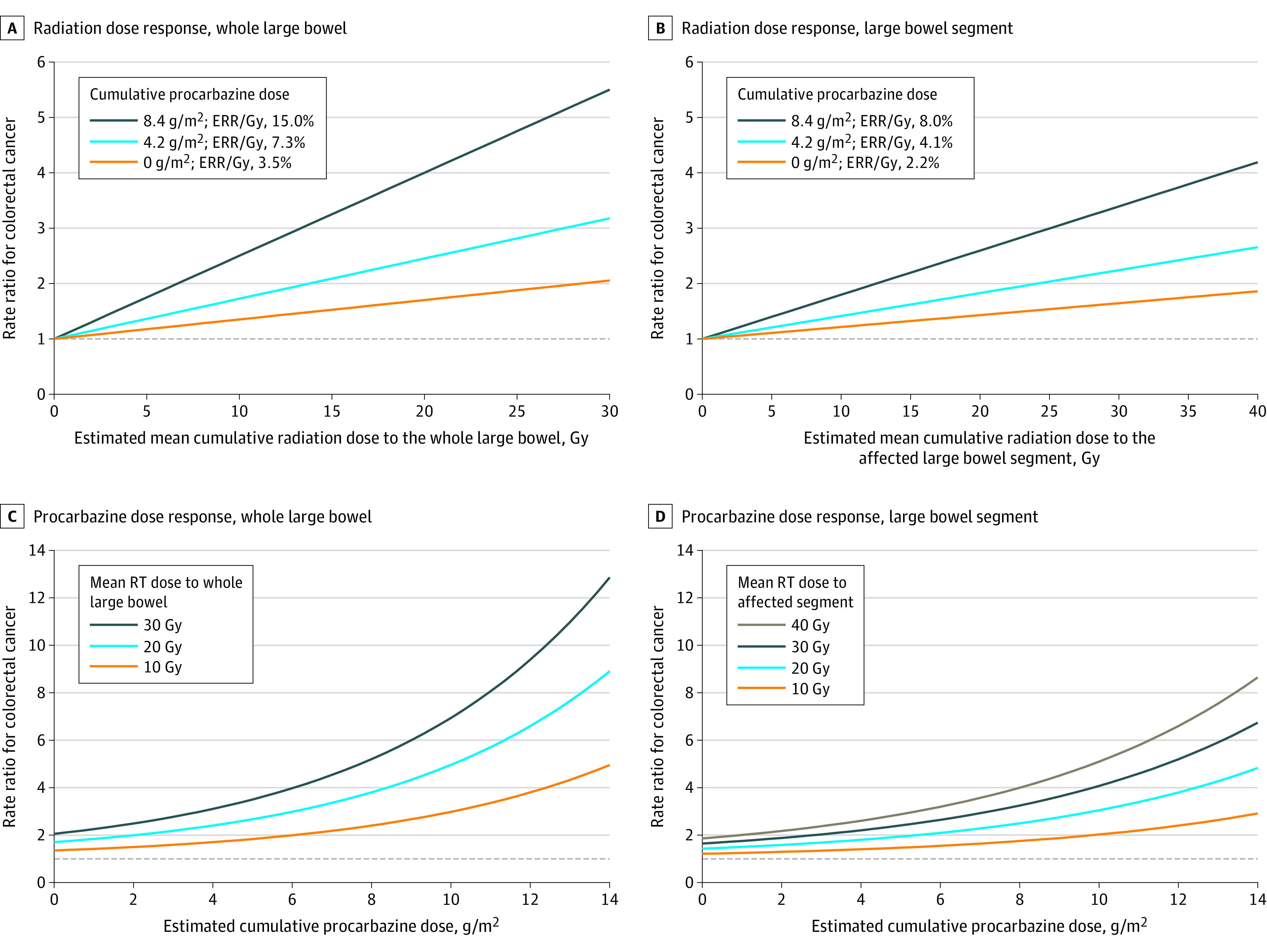
The Modeled Radiation Dose-Response Association for Colorectal Cancer After Hodgkin Lymphoma Including Effect Modification These models represent the radiation dose-response for mean radiation dose to the whole large bowel for incremental procarbazine dose levels (A); the radiation dose-response for mean radiation dose to the affected large bowel segment (matched segment for controls) for incremental procarbazine dose levels (B); the procarbazine dose-response for incremental radiation doses to the whole large bowel (C); and the procarbazine dose-response for incremental radiation doses to the affected large bowel segment (D). Rate ratios in panels A and B were obtained by evaluating rate ratios for procarbazine dose values using the formulas and keeping the radiation dose continuous. Rate ratios in panels C and D were obtained by evaluating rate ratios for radiation dose values using the formulas and keeping the cumulative procarbazine dose continuous. Patients who did not receive any radiation as part of HL treatment had a rate ratio for colorectal cancer of 1. Note that the radiation dose range differs for dose to the whole colon (up to 30 Gy) and dose to the affected segment (up to 40 Gy). ERR refers to excess rate ratio; g/m^2^, grams per meter squared; Gy, gray; HL, Hodgkin lymphoma; and RT, radiation therapy.

The radiation dose-response association was modified by cumulative procarbazine dose. A model with effect modification by continuous procarbazine dose had the lowest AIC (eTable 7 in Supplement 1). For radiation dose to the whole large bowel, the ERR/Gy was 3.5% (95% CI, 0.4%-12.6%) for patients who did not receive procarbazine. For each g/m^2^ increase in procarbazine dose, the ERR/Gy increased 1.19-fold (95% CI, 1.06-1.33-fold), with an ERR/Gy of 15.0% for patients who received 8.4 g/m^2^ procarbazine (Figure 3). For radiation dose to the affected segment, the ERR/Gy was 2.2% (95% CI, 0.2%-8.6%) for patients who did not receive procarbazine, and increased 1.17-fold (95% CI, 1.03-1.33-fold) for each g/m^2^ increase in procarbazine dose, with an ERR/Gy of 8.0% for patients treated with 8.4 g/m^2^ procarbazine (Figure 3). In a sensitivity analysis excluding patients who received 19.8 g/m^2^ or more procarbazine, the strength of effect modification by procarbazine dose did not change: 1.20-fold increase in ERR/Gy per g/m^2^ increase in procarbazine dose for both radiation dose to the whole large bowel and the affected segment.

For radiation dose to the whole large bowel, sex, age at HL diagnosis, time since first HL treatment, and receipt of anthracyclines did not modify the dose-response association (eTable 8 in Supplement 1). The dose-response association for radiation dose to the affected segment was modified by sex, with a higher ERR/Gy for females (ERR/Gy 39.1% vs 3.5% for males; eTable 9 in Supplement 1). Receipt of pelvic RT significantly increased the RR of colorectal cancer in women, but not in men (no pelvic RT vs pelvic RT; RR females, 12.0; 95% CI, 2.6-56.4; RR males, 1.6; 95% CI, 0.8-3.0), and the ERR/Gy was no longer modified by sex when patients who received pelvic RT were excluded.

## Discussion

This study is, to the best of our knowledge, the first study to demonstrate an association between therapeutic radiation dose to the large bowel and colorectal cancer. This association became stronger with increasing procarbazine dose, with highest rates for patients who received both a high radiation dose to the large bowel and a high cumulative procarbazine dose. For patients not treated with procarbazine, the ERRs/Gy for dose to the whole large bowel and the affected segment were 3.5% and 2.2%, respectively. In patients treated with 8.4 g/m^2^ procarbazine, ERRs/Gy for dose to the whole large bowel and the affected segment increased to 15.0% and 8.0%, respectively. When accounting for procarbazine, the model including whole large bowel dose had a lower (ie, better) AIC than the model with affected segment dose. Missing information on exact tumor location, use of representative rather than patient-specific CT data sets, and possible inaccuracy in dose reconstruction techniques and anatomical delineation of bowel segments may have affected accuracy of the segment dose model.

The overall ERRs/Gy for colorectal cancer were 9.2% for radiation dose to the whole large bowel and 7.5% for dose to the affected segment, respectively. These ERRs include the additional increase in risk associated with procarbazine exposure rather than showing the association of RT alone and are reported to allow for comparison with other studies not considering the effect-modification or confounding variables.

A previous study in a cohort of women treated for cervical cancer was unable to derive a radiation dose-response association with colorectal cancer risk.^29^ To our knowledge, the only other radiation dose-response association with colorectal cancer was derived from the Life Span Study in Japanese atomic bomb survivors. The ERRs/Gy findings in the present study are much lower than the other study’s^30^ ERR per Sievert of 39.5% (90% CI, 11.3%-72.5%). This is consistent with dose-response findings for other second solid cancers after radiation where the ERR/Gy is typically 5 to 10 times higher among atomic bomb survivors than among patients receiving therapeutic radiation.^31,32^ This difference is probably owing to the different radiobiologic effects from a single whole-body dose of radiation compared with more targeted, higher-dose, fractionated exposures.^31^

We observed that procarbazine dose modified the radiation dose-response association. Given that chemotherapeutic agents were routinely administered in combination with other therapy among our study cohort, we cannot be certain that the increased colorectal cancer rate was solely due to procarbazine. However, procarbazine is a known carcinogen,^33^ and being an alkylating agent, it has been shown to cause DNA methylation after conversion to methyldiazonium.^34^ Procarbazine has been implicated in gastric carcinogenesis,^16,35,36^ and it may come into direct contact with the colon mucosa after oral administration.^36^ Therefore, it may reasonably be considered to be the causative agent. Previous studies of gastric and pancreatic cancer after treatment for HL or childhood cancer have suggested a more than additive interaction between procarbazine and RT.^12,16,17,35,37,38,39^ The biologic mechanisms of interaction between radiation and procarbazine in carcinogenesis are not completely understood, but in vitro studies suggest a number of plausible mechanisms for synergistic effects in disrupting normal DNA repair and cell cycle progression.^40,41^ Although most carcinogenic substances have no threshold dose, we cannot exclude the possibility that procarbazine only increases colorectal cancer rate above a certain dose. However, because the present study had limited power to distinguish a possible threshold dose, cumulative procarbazine dose was modeled as a continuous variable.

Previous investigations^42,43,44,45^ have suggested that estrogens may be protective against colorectal cancer development; women using hormone replacement therapy have been shown to have a significantly lower risk of colorectal cancer compared with nonusers. High-dose procarbazine or pelvic radiation may induce early menopause,^46,47^ shortening the lifetime exposure of female patients to estrogen and perhaps explaining why our findings showed that the radiation dose-response associations differed by sex. More data are needed, however, because the number of female patients with colorectal cancer (n = 24) was small and the confidence intervals were wide. Age at HL diagnosis did not modify the radiation dose-response association, indicating that patients were at increased risk regardless of their age.

### Strengths and Limitations

The strengths of this study were the large number of cases, long-term follow-up of patients (median, 26.1 years), detailed RT and chemotherapy data, and individual reconstruction of radiation doses to the whole large bowel and large bowel segments for all cases and controls. Limitations included incomplete information on cumulative procarbazine dose and body surface area; therefore, we used procarbazine dose in g/m^2^ estimated from typical doses administered per cycle. Given that oral procarbazine may come into direct contact with the colon mucosa,^36^ cumulative procarbazine dose in grams may be more biologically relevant. Furthermore, representative rather than patient-specific CT data sets were used, so individual differences in colon shape, size, and location could not be considered. This was particularly relevant for patients who underwent a splenectomy as part of HL treatment (approximately 30%) because splenectomy may leave the position of the colon altered. Previous work by our group has shown that, using our reconstruction method, the effect of systematic uncertainty on the dose-response association is less than 10%, and the effect of random uncertainty is small, except at the highest radiation doses.^48^ Lastly, we used physical radiation dose, rather than biologically equivalent dose in calculations. The latter was used to account for fraction size variation in a previous study of radiation-related risk of valvular heart disease,^49^ but the effect of fraction size on the development of secondary malignant tumors after RT is not yet well quantified, and the α/β ratio for colorectal cancer as a secondary tumor has not been estimated yet.^50^ Regardless, using the physical dose allowed for comparison with previous studies on secondary cancer risk after RT.^15,51,52^

## Conclusions

This nested case-control study of 316 HL survivors suggests a dose-response association of colorectal cancer risk with RT for HL. Although HL treatments have evolved considerably during the recent decades, with fewer patients receiving subdiaphragmatic RT or high-dose procarbazine, these findings remain relevant and important for clinicians treating patients for HL in the modern era. This evidence enables individualized estimation of colorectal cancer risk and selection of the optimal treatment strategy for patients who are treated with subdiaphragmatic RT (eg, for diffuse large B-cell lymphoma) or with procarbazine, eg, as part of the BEACOPP regimen (bleomycin, etoposide, doxorubicin, cyclophosphamide, vincristine, procarbazine, prednisone). These findings also emphasize the need for clinicians to identify HL survivors previously treated with subdiaphragmatic RT and procarbazine for whom colorectal cancer screening should be considered.^38,53,54^ Also, given their elevated risk of colorectal cancer, these patients should receive guidance on minimizing risk by avoiding associated risk factors, such as smoking, alcohol use, and being overweight. These findings have an important role in addressing the association of the late effects of HL treatment on the healthy life expectancy of HL survivors.

## Notes

Supplement 1. **eTable 1**. Selection process of cases and controls **eTable 2.** Distribution of the factors used for selecting matched controls for Hodgkin lymphoma survivors who developed colorectal cancer
eMethods.
**eTable 3.** Selection of the representative CT library **eFigure 1.** Radiotherapy simulation film and reconstructed plan **eFigure 2. **Position of the large bowel segments in relation to the reconstructed radiotherapy fields **eFigure 3.** Large bowel anatomy in each of the representative CTs **eTable 4.** Distribution of the patient- and treatment-related factors of HL survivors who developed colorectal cancer and matched controls **eTable 5.** Patient characteristics and risk factors by radiation field **eTable 6. **Risk of CRC after HL in relation to procarbazine dose and radiation dose to the affected large bowel segment and whole large bowel **eFigure 4. **The modeled overall radiation dose-response relationship for colon cancer following Hodgkin lymphoma with mean radiation dose to the colon **eTable 7. **Overview of model specifications **eTable 8. **Median dose and excess rate ratios for radiation to the whole large bowel according to patient- and treatment-related factors **eTable 9. **Median mean dose and excess rate ratios for radiotherapy to the affected large bowel segment according to patient- and treatment-related factors

Supplement 2.
Data Sharing Statement
