# Supplementary material for: Association of Radiation and Procarbazine Dose With Risk of Colorectal Cancer Among Survivors of Hodgkin Lymphoma
Source: JAMA Oncol. 2023 Feb 2;9(4):481–9. doi: 10.1001/jamaoncol.2022.7153 (PMC9896374; doi:10.1001/jamaoncol.2022.7153)
Supplement: Supplement 1. — eTable 1. Selection process of cases and controls eTable 2. Distribution of the factors used for selecting matched controls for Hodgkin lymphoma survivors who developed colorectal cancer eMethods. eTable 3. Selection of the representative CT library eFigure 1. Radiotherapy simulation film and reconstructed plan eFigure 2. Position of the large bowel segments in relation to the reconstructed radiotherapy fields eFigure 3. Large bowel anatomy in each of the representative CTs eTable 4. Distribution of the patient- and treatment-related factors of HL survivors who developed colorectal cancer and matched controls eTable 5. Patient characteristics and risk factors by radiation field eTable 6. Risk of CRC after HL in relation to procarbazine dose and radiation dose to the affected large bowel segment and whole large bowel eFigure 4. The modeled overall radiation dose-response relationship for colon cancer following Hodgkin lymphoma with mean radiation dose to the colon eTable 7. Overview of model specifications eTable 8. Median dose and excess rate ratios for radiation to the whole large bowel according to patient- and treatment-related factors eTable 9. Median mean dose and excess rate ratios for radiotherapy to the affected large bowel segment according to patient- and treatment-related factors [file jamaoncol-e227153-s001.pdf]

Supplemental Online Content

Geurts YM, Shakir R, Ntentas G, et al. Association of radiation and procarbazine dose with risk of colorectal cancer among survivors of Hodgkin lymphoma. *JAMA Oncol.* Published online February 2, 2023. doi:10.1001/jamaoncol.2022.7153

eTable 1 . Selection process of cases and controls

eTable 2. Distribution of the factors used for selecting matched controls for Hodgkin lymphoma survivors who developed colorectal cancer

eMethods.

eTable 3. Selection of the representative CT library

eFigure 1. Radiotherapy simulation film and reconstructed plan

eFigure 2. Position of the large bowel segments in relation to the reconstructed radiotherapy fields

eFigure 3. Large bowel anatomy in each of the representative CTs

eTable 4. Distribution of the patient- and treatment-related factors of HL survivors who developed colorectal cancer and matched controls

eTable 5. Patient characteristics and risk factors by radiation field

eTable 6. Risk of CRC after HL in relation to procarbazine dose and radiation dose to the affected large bowel segment and whole large bowel

eFigure 4. The modeled overall radiation dose-response relationship for colon cancer following Hodgkin lymphoma with mean radiation dose to the colon

eTable 7. Overview of model specifications

eTable 8. Median dose and excess rate ratios for radiation to the whole large bowel according to patient- and treatment-related factors

eTable 9. Median mean dose and excess rate ratios for radiotherapy to the affected large bowel segment according to patient- and treatment-related factors

This supplemental material has been provided by the authors to give readers additional information about their work.

eTable 1. Selection process of cases and controls

	Cases<br>No. (%)	Controls,<br>No. (%)
Initially selected	83	277
Ineligible	5 (6.0)	24 (8.7)
Other pathology	3 (3.6)	
Linkage error	2 (2.4)	
Medical record lost/incomplete		8 (2.9)
Subdiaphragmatic RT for other cancer		5 (1.8)
Deceased before cut-off		7 (2.5)
Lost-to-follow-up before cut-off		4 (1.4)
Selected as control, case excluded		15 (5.4)
Total excluded	5 (6.0)	39 (14.1)
Total included	78 (94.0)	238 (85.9)
Duplicate controls		
Case as control		
One time		1 (0.4)
Control as control		
One time		100 (42.0)
Two times		27 (11.3)
Three times		16 (6.7)
Four times		5 (2.1)
Five times		2 (0.8)
Six times		1 (0.4)
Total number of unique individuals	78 (100.0)	151 (63.4)

Patients were eligible for inclusion if they had first been treated for Hodgkin lymphoma between 1965 and 2000, when they were between 15 and 50 years of age, and had survived for at least 5 years after receiving treatment. If radiotherapy to the abdomen or the pelvis was given for reasons other than Hodgkin lymphoma, the patient was ineligible for inclusion in the case-control study.
No., number; RT, radiotherapy

© 2023 Geurts YM et al. *JAMA Oncol.*

eTable 2. Distribution of the factors used for matching (sex, year of HL diagnosis, age at HL diagnosis), follow-up time and age at end of follow-up of HL survivors who developed colorectal cancer and matched controls

Matching factors	Cases (n=78) No. (%)	Controls (n=238) No. (%)	Unique controls (n=151) No. (%)
Sex			
Male	54 (69.2)	167 (70.2)	95 (62.9)
Female	24 (30.8)	71 (29.8)	56 (37.1)
Period of HL diagnosis			
1964-1976	35 (44.9)	100 (42.0)	54 (35.8)
1977-1988	31 (39.7)	99 (41.6)	68 (45.0)
1989-2000	12 (15.4)	39 (16.4)	29 (19.2)
Age at HL diagnosis, years			
15-24	21 (26.9)	71 (29.8)	40 (26.5)
25-34	24 (30.8)	73 (30.7)	39 (25.8)
35-50	33 (42.3)	94 (39.5)	72 (47.7)
Median (IQR)	33.1 (24.2-41.7)	33.0 (24.0-41.3)	34.7 (24.7-42.6)
Follow-up time, years[a]			
5-14	15 (19.2)	38 (16.0)	31 (20.5)
15-24	23 (29.5)	76 (31.9)	49 (32.5)
25-34	29 (37.2)	92 (38.7)	51 (33.8)
≥35	11 (14.1)	32 (13.5)	20 (13.3)
Median (IQR)	25.7 (18.2-31.6)	26.1 (18.3-31.1)	23.5 (16.2-30.5)
Age at end of follow-up, years[b]			
30-50	14 (18.0)	41 (17.2)	22 (14.6)
50-64	49 (62.8)	153 (64.3)	100 (66.2)
65-80	15 (19.2)	44 (18.5)	29 (19.2)
Median (IQR)	59.1 (51.9-63.3)	58.8 (53.0-62.6)	59.5 (53.0-62.8)

[a] Time from HL treatment to colorectal cancer (cases) or cut-off date (controls)
[b] Age at colorectal cancer diagnosis (cases) or cut-off date (controls)
HL, Hodgkin lymphoma; IQR, interquartile range; No., number

© 2023 Geurts YM et al. *JAMA Oncol.*

eMethods

Matching and selection of controls

Cases and controls were ineligible for inclusion if information from their medical record was incomplete or if radiotherapy to the abdomen or the pelvis was given for reasons other than Hodgkin lymphoma (HL, eTable 1). Controls also had to stay alive and remain free of colorectal cancer for a time interval equal to the interval from date of HL diagnosis to the date of colorectal cancer for the matched case (cut-off). Controls were selected by incidence density sampling: all survivors remained eligible as controls until they either had a case-defining event or were censored.[1] Survivors could be a control for more than one case.

For each case with colorectal cancer (CRC), up to five controls were selected from the cohort. Controls were individually matched to cases on sex, age at HL diagnosis (within one year) and date of HL diagnosis (within three years). Matching criteria were relaxed up to a maximum difference of three years for age at HL diagnosis and four years for date of HL diagnosis to enable selection of controls. Controls were ranked based on the matching criteria from closest (most optimal) control to the furthest (least optimal) control. Insufficient follow-up time was not a reason to exclude a control a priori, as updated information on this variable could be collected from the medical record. For the five most optimal controls for each case, we checked whether they met the eligibility criteria, especially the interval criterion, using data from the medical files. For many cases, there were less than five eligible controls available. To increase power, we kept controls ranked fourth or fifth when they fitted the eligibility criteria and used a variable matching ratio in analysis.

Treatment

All included HL survivors had been treated with radiotherapy and/or chemotherapy, the majority according to European Organization for Research and Treatment of Cancer Lymphoma Group protocols for primary treatment.[2] Briefly, in the 1960s, RT was usually delivered with cobalt-60 and sometimes with orthovoltage; from the 1970s onwards, linear accelerators were used. Individual blocks were used to shield normal tissues where possible. Patients usually received 40 Gray (Gy) when they were treated with RT alone, and 30-36 Gy when they also received chemotherapy, in both cases with 1.5-2.0 Gy fractions. Extended-field RT including mantle, para-aortic (±spleen/splenic hilum) and iliac and inguinal fields, was commonly given until the late 1980s. Following this, involved-field RT was gradually introduced.

In the 1960s-70s, monotherapy with vinblastine was the most frequently prescribed chemotherapy. From the 1970s to the late 1980s, chemotherapy mainly consisted of MOPP

© 2023 Geurts YM et al. *JAMA Oncol.*

(mechlorethamine, vincristine, procarbazine, prednisone). Following this, anthracycline-containing regimens such as alternating MOPP and ABVD (MOPP/doxorubicin, bleomycin, vinblastine, dacarbazine) and hybrid MOPP/ABV (MOPP/doxorubicin, bleomycin, vinblastine) were introduced as primary treatment.

Development of a representative computed tomography library

The Eclipse treatment planning system (TPS) (Version 13.0.28, Varian Medical Systems, Palo Alto, CA) was used to retrospectively reconstruct the radiotherapy fields delivered to cases and controls. Twelve representative computed tomography (CT) data sets (six female and six male) were chosen from a cohort of 66 recently treated Hodgkin lymphoma (HL) patients (30 female and 36 male), based on body surface separation. These CTs were taken after two or three cycles of chemotherapy, and were therefore closest in time sequence to the measurements taken for radiotherapy planning in the historic cohort. Separation was measured as the distance between the anterior and posterior body surfaces on axial CT slices at the following points: upper neck, lower neck, mid-mediastinum, lower mediastinum, para-aortic and ileocecal. These points match the anatomical landmarks used in radiotherapy dose calculations for the cohort.

Separation at the same anatomical points was extracted from the radiotherapy prescription charts of patients in the cohort where available. The cohort was then split into tertiles, separate for each sex, based on their mid-mediastinal separation and again based on their para-aortic separation. Representative CTs were chosen from the modern cohort such that they most closely matched the median separation for each tertile of the historic cohort (eTable 3). The chosen CT was reviewed to ensure acceptable image quality, and absence of major anatomical variations or persistent large-volume lymphoma. The final representative CT library contains 12 full-body CT scans: three each for supradiaphragmatic dose-reconstruction for females and males, and three each for subdiaphragmatic dose-reconstruction for females and males.

All the abdominal-pelvic organs were contoured as per published guidelines[3,4] by a clinical oncologist (RS or DC) and counter-checked by the other. Where there was any doubt or disagreement, the contours were checked by a radiologist. The bowel was outlined to include the external bowel wall plus contents.

For each individual in the cohort who was treated with radiotherapy, a representative CT was chosen based on his or her separation at either the para-aortic or mid-mediastinal level. Individuals treated with both supra- and subdiaphragmatic fields had the representative CT chosen that most closely matched the separation at the para-aortic level, as dose to abdominal-pelvic organs was the primary focus of this study. Individuals treated with only supra- or subdiaphragmatic

© 2023 Geurts YM et al. *JAMA Oncol.*

radiotherapy had the representative CT chosen based on only the mid-mediastinal or para-aortic separation, respectively.

eTable 3. Selection of the representative computed tomography library

Supradiaphragmatic	Range of mediastinal separation in historic cohort (cm)	Median mediastinal separation in historic cohort (cm)	Separation of chosen representative CT from modern cohort (cm)
Males			
Group 1	17.0 – 20.3	18.65	20.1 (lowest suitable)
Group 2	20.4 – 23.7	22.05	22.0
Group 3	23.8 – 27.1	25.45	25.5
Females			
Group 1	16.0 – 18.6	17.30	18.3 (lowest suitable)
Group 2	18.7 – 21.3	20.00	21.0[a]
Group 3	21.4 – 24.0	22.70	23.5
Subdiaphragmatic	Range of para-aortic separation in historic cohort (cm)	Median para-aortic separation in historic cohort (cm)	Separation of chosen representative CT from modern cohort (cm)
Males			
Group 1	16.0 – 19.6	17.80	19.1 (lowest suitable)
Group 2	19.7 – 23.3	21.50	21.4
Group 3	23.4 – 27.1	25.25	25.3
Females			
Group 1	11.5 – 16.1	13.80	14.6 (lowest suitable)
Group 2	16.2 – 20.8	18.50	18.6
Group 3	20.9 – 25.5	23.20	23.8

[a] Chosen to ensure that the 'small' and 'medium' representative CTs were not too close in separation.
cm, centimeter; CT, computed tomography; n, number

Treatment reconstruction

Treatment planning variables, including prescribed dose, fractionation regime, beam arrangement, beam energy, source-to-skin distance, field size, and field shielding were extracted from the patients' original radiotherapy prescription cards. This information was used in combination with the original simulation films to individually reconstruct the treatments for 72 cases and 131 controls, blinded to whether the individual was a case or control. The relationship of block borders to anatomic landmarks, shown on the simulation films, was used as a guide to reconstruct the field shielding in the treatment planning system. An example of a reconstruction is given in eFigure 1.

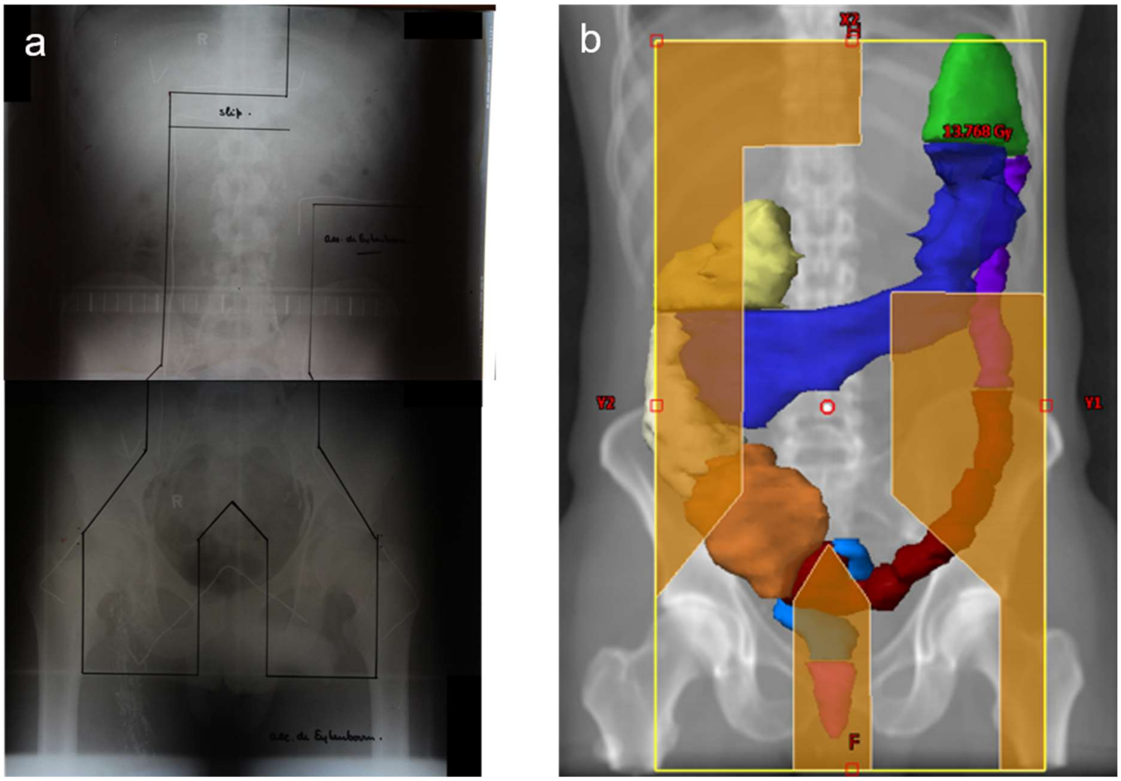

eFigure 1. An example of (a) a radiotherapy simulation film and (b) reconstructed radiotherapy plan

The original simulation films (a) were used to reconstruct the radiotherapy fields on a digitally reconstructed radiograph from the representative CT data set (b), and block borders (black in a, orange in b) matched to anatomic landmarks.

For patients who were treated with a combination of mediastinal and subdiaphragmatic fields, common practice was to include a slip zone between the inferior border of the mediastinal field and the superior border of the subdiaphragmatic field (typically an inverted Y or para-aortic field). These borders were moved up or down during treatment; the aim of this was to minimize the effect of hot or cold spots at the junction of the two fields. Where this technique was used, this was incorporated into the individual reconstruction.

When treating patients with bulky mediastinal disease, common practice was to start radiotherapy with a wide mediastinal field and then narrow it once the disease started to respond and shrink. Where this was the case, and when the simulation films indicated the borders of the wide and narrow fields, this was also incorporated into the individual reconstruction.

Where simulation films were not available (26 cases and 34 controls, total 232 fields), fields were reconstructed using multiple other sources of information. The field type and size were extracted from the radiotherapy prescription, along with information from any diagrams or dose calculations contained within the prescription. This information was used in combination with the

© 2023 Geurts YM et al. *JAMA Oncol.*

fields used in other patients treated at the same treatment center at a similar time to the individual in question.

For 25 patients, there was no information on fractionation or beam energy for primary and/or relapse treatment, and this was imputed from other individuals of similar size (separation) treated at the same center with the same field type in the same period. For five cases and six controls, there was no information on prescribed dose for primary and/or relapse treatment, this was again imputed from other individuals treated at the same center with the same field types in the same period. For one case and one control, information on radiation dose, field and energy was not sufficient to impute a radiation dose, these two patients were included in the unknown radiation dose category. A sensitivity analysis was done to determine the effect of imputing missing subdiaphragmatic RT doses (three cases and three controls); results are presented in the main article.

Seven patients were treated with orthovoltage fields (30 fields, 3.4% of patients, 2.9% of fields) which we were unable to reconstruct using the Eclipse TPS. However, these fields were typically used as boost to the groins or axilla, for example following megavoltage or cobalt radiotherapy, and as such, the dose to the colon or rectum would have been negligible. These fields were therefore omitted. Patients were treated with between one and 20 fields each (median four); 1027 fields were reconstructed for 203 unique patients (73 cases, 131 controls).

Dose calculation

The anisotropic analytical algorithm (AAA) photon dose calculation model in the Eclipse TPS was used to calculate the dose distributions for patients who received radiotherapy using a linear accelerator (170 patients [83.3%], 919 fields [89.5%]). The pencil beam convolution (PBC) algorithm was used for patients who received radiotherapy using cobalt-60 (23 patients [11.3%], 63 fields [6.1%]). AAA results in more accurate dose distributions compared to PBC, because it accounts for lateral electron transfer, which is especially relevant in heterogeneous and low density tissue (such as lung)[5]. However, as cobalt-60 is a historic treatment, there is no AAA model available to use in combination with a cobalt-60 beam model in the Eclipse TPS. Given that our focus is on doses to the large bowel, which is a more homogeneous and higher density tissue than lung, it is likely that the impact of using PBC rather than AAA is extremely small. All radiotherapy TPS's are unreliable at evaluating out-of-field dose, and therefore low-dose estimates (less than 1Gy) are unlikely to be accurate.[6] This is unlikely to influence our dose-response relationship as the lowest dose category starts at 1.0 Gy, and the reference category was those not treated with radiotherapy or who received less than 1.0 Gy to the affected colon segment. Once the dose from each field had been calculated, the dose distributions from all fields were summed for each

© 2023 Geurts YM et al. *JAMA Oncol.*

individual, and the mean doses to each segment of colon and rectum were extracted: cecum, ascending colon, hepatic flexure, transverse colon, splenic flexure, descending colon and sigmoid colon. The location of these structures in relation to the five main types of radiotherapy fields is shown in eFigure 2. For three cases exact information on the location of the subsequent colon tumor was not available; for these cases (and their corresponding controls) doses were estimated for the right colon (one case, CRC in the right part of colon) or the whole colon (two cases, unknown CRC location).

© 2023 Geurts YM et al. *JAMA Oncol.*

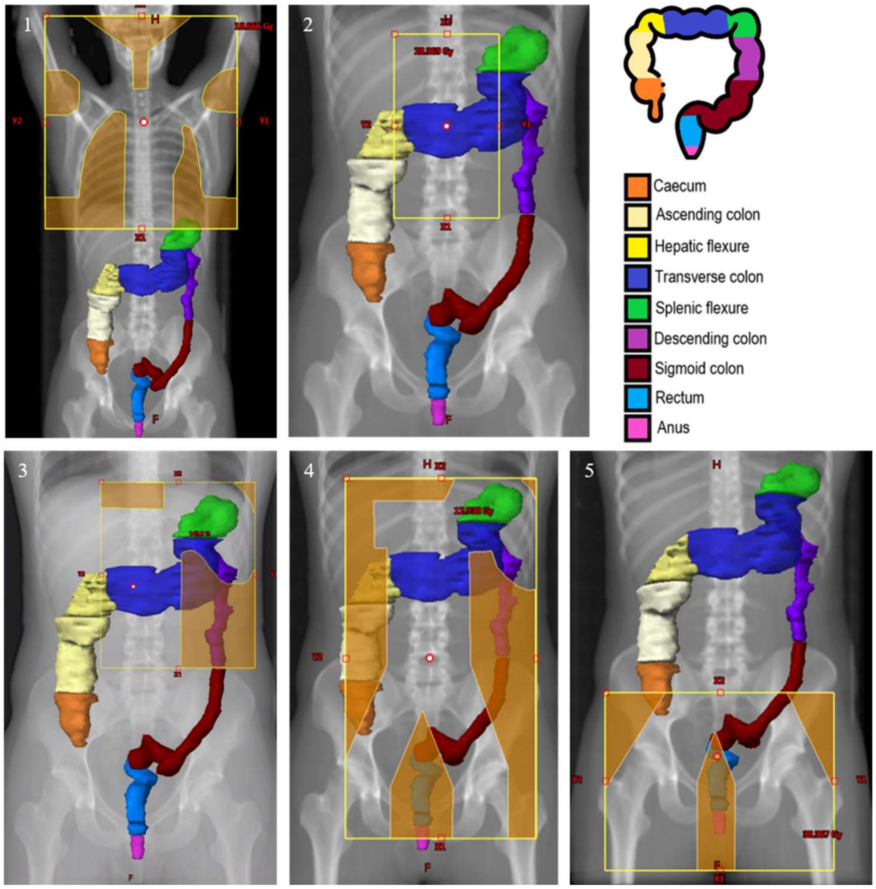

eFigure 2. Position of the colon segments in relation to the reconstructed radiotherapy fields

Field borders are indicated with thin yellow lines, with shielding from blocks shown by the shaded orange regions

1. Supradiaphragmatic (mantle) field, treating the mediastinal, hilar, supraclavicular, cervical, infraclavicular and axillary lymph nodes
2. Para-aortic field without spleen (these fields did not use additional shielding)
3. Para-aortic field with spleen
4. Para-aortic with iliac field (Inverted Y) and spleen
5. Iliac field

© 2023 Geurts YM et al. *JAMA Oncol.*

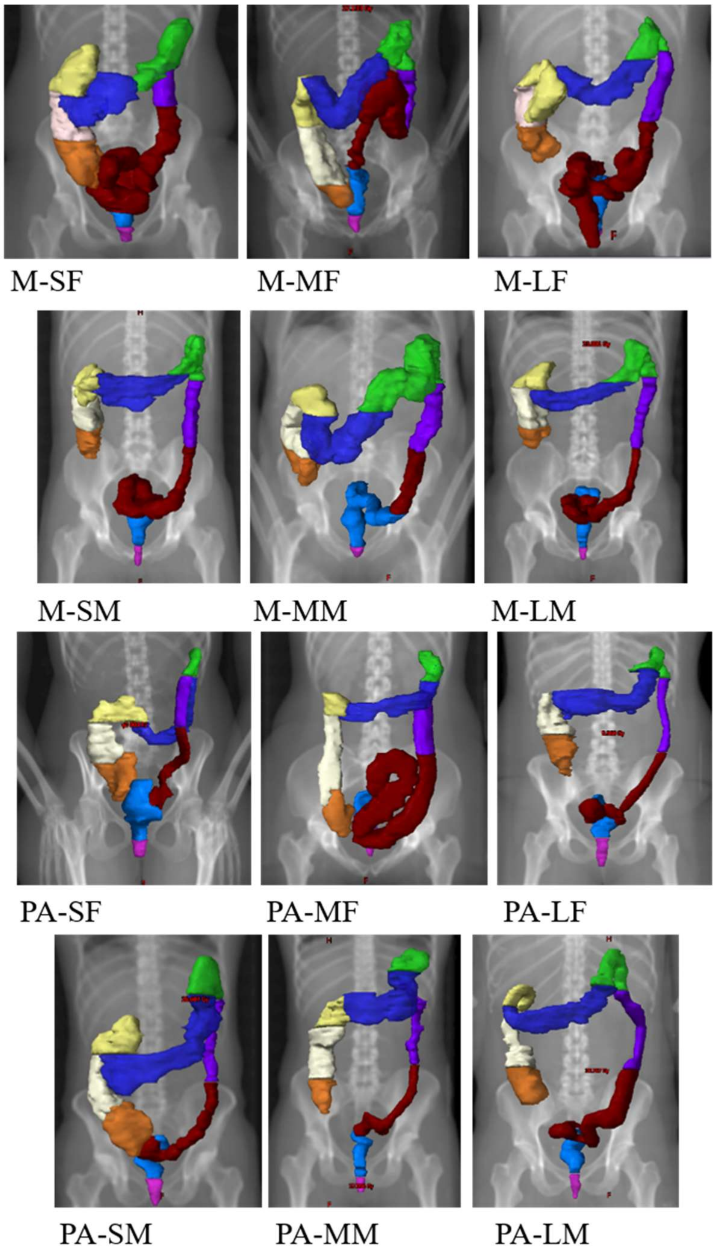

eFigure 3. Large bowel anatomy in each of the representative CT scans

Twelve representative CT data sets were used: six male and six female. For each sex, three CT data sets were matched on para-aortic separation and used to reconstruct subdiaphragmatic radiotherapy fields (PA-SF, PA-MF, PA-LF, PA-SM, PA-MM, PA-LM). The remaining three CT data sets for each sex were matched on mediastinal separation and used to reconstructed supradiaphragmatic radiotherapy fields. If patients had been treated with both supra- and subdiaphragmatic radiotherapy fields, the para-aortic CT data sets were used to reconstruct all radiotherapy fields so that the radiation dose to the colon from all the fields combined could be estimated.

CT, computed topography; M, mediastinum; SF, small female; MF, medium female; LF, large female; SM, small male; MM, medium male; LM, large male; PA, para-aortic.

© 2023 Geurts YM et al. *JAMA Oncol.*

References

1. Lubin JH, Gail MH. Biased selection of controls for case-control analyses of cohort studies. Biometrics. 1984;40(1):63-75.
2. Eghbali H, Raemaekers J, Carde P. The EORTC strategy in the treatment of Hodgkin's lymphoma. Eur J Haematol Suppl. 2005(66):135-40.
3. Gay HA, Barthold HJ, O'Meara E, Bosch WR, El Naqa I, Al-Lozi R, et al. Pelvic normal tissue contouring guidelines for radiation therapy: a Radiation Therapy Oncology Group consensus panel atlas. Int J Radiat Oncol Biol Phys. 2012;83(3):e353-62.
4. Jabbour SK, Hashem SA, Bosch W, Kim TK, Finkelstein SE, Anderson BM, et al. Upper abdominal normal organ contouring guidelines and atlas: a Radiation Therapy Oncology Group consensus. Pract Radiat Oncol. 2014;4(2):82-9.
5. Rønde HS, Hoffmann L. Validation of Varian's AAA algorithm with focus on lung treatments. Acta Oncol. 2009;48(2):209-15.
6. Howell RM, Scarboro SB, Kry SF, Yaldo DZ. Accuracy of out-of-field dose calculations by a commercial treatment planning system. Phys Med Biol. 2010;55(23):6999-7008.

© 2023 Geurts YM et al. *JAMA Oncol.*

**eTable 4. Distribution of the patient- and treatment-related factors of HL survivors who developed colorectal cancer, and matched controls[a]**				
Patient- and treatment characteristics	Cases No. (%)	Controls No. (%)	Rate ratio (95% CI)	$P_{heterogeneity}$[b]
HL stage				>0.5
I	24 (30.8)	57 (24.0)	Ref.	
II	32 (41.0)	103 (43.3)	0.7 (0.4-1.4)	
III	14 (18.0)	43 (18.1)	0.8 (0.4-1.6)	
IV	8 (10.3)	34 (14.3)	0.5 (0.2-1.3)	
Unknown	0 (0.0)	1 (0.4)	-	
Treatment category				0.01
Primary CT only	4 (5.1)	28 (11.8)	Ref.	
Primary RT only	18 (23.1)	85 (35.7)	1.8 (0.6-5.9)	
Primary CT and RT	27 (34.6)	71 (29.8)	3.4 (1.0-10.9)	
Primary and relapse CT or RT only	2 (2.6)	7 (2.9)	3.0 (0.4-20.9)	
Primary and relapse CT and RT	27 (34.6)	47 (19.8)	5.2 (1.5-17.3)	
Radiotherapy field				0.003
No subdiaphragmatic RT	30 (38.5)	141 (59.2)	Ref.	
Para-aortic (±spleen)	16 (20.5)	39 (16.4)	1.9 (0.9-4.0)	
Inverted Y or iliac (±spleen)	32 (41.0)	58 (24.4)	2.8 (1.5-5.1)	
Splenectomy				>0.5
No	51 (65.4)	165 (69.3)	Ref.	
Yes	26 (33.3)	73 (30.7)	1.1 (0.6-2.0)	
Unknown	1 (1.3)	0 (0.0)	-	
Any anthracyclines				>0.5
No	57 (73.1)	172 (72.3)	Ref.	
Yes	21 (26.9)	66 (27.7)	1.0 (0.5-2.0)	
Any alkylating agents				0.08
No	25 (32.0)	103 (43.3)	Ref.	
Yes	53 (68.0)	135 (56.7)	1.6 (0.9-2.9)	
Any vinca-alkaloids				
No	20 (25.6)	87 (36.6)	Ref.	0.09
Yes	58 (74.4)	151 (63.4)	1.6 (0.9-2.9)	
Any procarbazine				0.05
No	27 (34.6)	113 (47.5)	Ref.	
Yes	51 (65.4)	125 (52.5)	1.7 (1.0-2.9)	
Procarbazine dose, g/m^2 [c]				0.005
No procarbazine	27 (34.6)	113 (47.5)	Ref.	
1.0 to 5.6 (median 4.2 g/m^2)	12 (15.4)	52 (21.9)	0.7 (0.3-1.8)	
>5.6 (median 8.4 g/m^2)	39 (50.0)	73 (30.7)	2.4 (1.3-4.5)	
Chemotherapy regimen				0.05
No chemotherapy	20 (25.6)	87 (36.6)	Ref.	
MOPP/MVPP	23 (29.5)	44 (18.5)	2.3 (1.1-4.8)	
MOPP-ABV hybrid	9 (11.5)	26 (10.9)	1.6 (0.6-4.5)	
MOPP-ABVD (alternating)	0 (0.0)	14 (5.9)	-	
MOPP-like[d]	8 (10.3)	24 (10.1)	1.5 (0.6-3.8)	
ABVD	2 (2.6)	7 (2.9)	1.5 (0.3-8.5)	
Vinblastine monotherapy	4 (5.1)	12 (5.0)	1.4 (0.4-4.8)	
Other[e]	12 (15.4)	24 (10.1)	2.2 (0.9-5.3)	

© 2023 Geurts YM et al. *JAMA Oncol.*

**Supplementary Table 4 continued.**				
Patient- and treatment characteristics	Cases No. (%)	Controls No. (%)	Rate ratio (95% CI)	$P_{heterogeneity}$[b]
Overweight at HL diagnosis[f]				>0.5
No	49 (62.8)	167 (70.2)	Ref.	
Yes	14 (18.0)	41 (17.2)	1.0 (0.5-2.1)	
Unknown	15 (19.2)	30 (12.6)	-	
Smoking status at HL diagnosis[f]				0.09
Never	24 (30.8)	80 (33.6)	Ref.	
Former	10 (12.8)	23 (9.7)	1.8 (0.7-4.7)	
Recent, light smoker	11 (14.1)	61 (25.6)	0.6 (0.3-1.5)	
Recent, moderate smoker	14 (18.0)	32 (13.5)	1.7 (0.7-3.9)	
Recent, heavy smoker	3 (3.9)	17 (7.1)	0.7 (0.2-2.6)	
Ever	13 (16.7)	22 (9.2)	2.3 (0.96-5.6)	
Unknown	3 (3.9)	3 (1.3)	-	
RT dose to affected segment and procarbazine dose[c,g, h]				0.004
<20 Gy and ≤4.2 g/m^2	28 (36.4)	125 (52.7)	Ref.	
<20 Gy and >4.2 g/m^2	25 (32.5)	73 (30.8)	1.5 (0.8-2.9)	
≥20 Gy and ≤4.2 g/m^2	8 (10.4)	22 (9.3)	1.9 (0.7-5.2)	
≥20 Gy and >4.2 g/m^2	16 (20.8)	17 (7.2)	4.7 (2.0-10.8)	

[a] Includes primary treatment and treatment for recurrence
[b] Unknown category not included in calculation of $P_{heterogeneity}$
[c] Assuming a procarbazine dose of 1.4 g/m^2 per cycle (14 days x 0.1 g/m^2 per day), 4.2 g/m^2 corresponds to three cycles and 8.4 g/m^2 to six cycles of MOPP. Other protocols (e.g. MOPP/ABV) include a procarbazine dose of 0.7 g/m^2 per cycle.
[d] Included in the MOPP-like chemotherapy category: MOPP+vinblastine (1 case, 6 controls), MOPP+cyclophosphamide (1 case, 4 controls), MOPP+chlorambucil (1 case, 1 control), MOPP+lomustine-OPP (2 controls), MOPP+lomustine (1 control), MOPP+mitoxine (1 case), MOPP+C-MOPP (1 case), MOPP+COPP (1 control), MOPP+CH1VPP (1 control), BCVPP (1 case, 2 controls), CCVPP (1 case), COPP (1 control), MOPP+cyclophosphamide+procarbazine (1 control), MOPP+mitoxine+procarbazine (1 case), MOPP+lomustine+vinblastine (1 control), MOPP+chlorambucil+vinblastine (1 control), MOPP+OPP+lomustine-OPP+vinblastine (1 control), MOPP+cyclophosphamide+mitoxine (1 control)
[e] The category "other" consists of: EBVP (1 case, 4 controls), MOPP/CHVmP (5 controls), MOPP/ABV+CBV+DHAP-VIM-DHAP (3 controls), MOPP/CVBP (2 controls), BEACOPP (2 controls), MOPP+ABVD+DHAP+CBV (2 controls), chlorambucil+procarbazine+vinblastine (1 case), procarbazine+vinblastine (1 case), MOPP-CHVmP+MOPP+CH1VPP+MOPP/ABV+CVP (1 case), COPP+ACOP+cyclophosphamide+bleomycin+lomustine (1 case), VAPP+BCVPP (1 case), MOPP+lomustine-OPP+ABVD (1 case), MOPP+CHOP+bleomycin+lomustine+vinblastine (1 case), MOPP/ABV+DHAP+CBV (1 case), MOPP/ABVD+MOPP/ABV (1 case), MOPP/ABV+ DHAP+BEAM (1 case), MOPP+CVB+lomustine+BCVPP+MOPP/ABV+DHAP-VIM-DHAP (1 case), cyclophosphamide+mitoxine+vincristine (1 control), chlorambucil+mitoxine+vinblastine (1 control), cyclophosphamide+vinblastine (1 control), ABVD+MOPP+PROMACE+CBV (1 control), MOPP/ABV+DHAP-VIM-DHAP+BEAM+Brentuximab (1 control), BCVPP+ABVD (1 control)
[f] An individual was considered overweight when he/she had a BMI ≥25 kg/m^2 or if there was indicated in the medical file that the patient was "too heavy" or "adipose". A total of 12 patients had a BMI ≥30 kg/m^2 at Hodgkin lymphoma diagnosis (12 controls).
[g] Patients who were recent cigarette smoker at diagnosis were further subdivided into smokers of less than 14 cigarettes per day or light smokers, 15 to 24 cigarettes per day or moderate smokers, ≥25 cigarettes per day or heavy smokers. Patients for whom it was unknown if they were recent or former smokers or for whom the number of cigarettes per day was unknown were classified as "ever smokers".

© 2023 Geurts YM et al. *JAMA Oncol.*

[g] Matched segment for controls
[h] There was no statistically significant additive (p=0.23) or multiplicative (p=0.47) interaction between radiation dose to the affected large bowel segment in categories and procarbazine dose in categories. P values were calculated using a likelihood ratio test
ABV, doxorubicin, bleomycin, vinblastine; ABVD, doxorubicin, bleomycin, vinblastine, dacarbazine; ACOP, doxorubicin, cyclophosphamide, vincristine, prednisone; BEACOPP, bleomycin, etoposide, doxorubicin, cyclophosphamide, vincristine, procarbazine, prednisone; BEAM, carmustine, etoposide, cytarabine, melphalan; BCVPP, carmustine, cyclophosphamide, vinblastine, procarbazine, prednisone; CBV, cyclophosphamide, carmustine, etoposide; CCVPP, lomustine, cyclophosphamide, vinblastine, procarbazine, prednisone; CH1VPP, chlorambucil, vinblastine, procarbazine, prednisone; CHOP, cyclophosphamide, doxorubicin, vincristine, prednisone; CHVmP, cyclophosphamide, doxorubicin, teniposide, prednisone; COPP, cyclophosphamide vincristine, procarbazine, prednisone; C-MOPP, cyclophosphamide vincristine, procarbazine, prednisone; CT, chemotherapy; CVB, cyclophosphamide, bleomycin, vinblastine; CVBP, carmustine, vincristine, bleomycin, prednisone; CVP, cyclophosphamide, etoposide, carboplatin; DHAP, dexamethasone, cytarabine, cisplatin; EBVP, epirubicine, bleomycine, vinblastine, prednisone; HL, Hodgkin lymphoma; MOPP, mechlorethamine, vincristine, procarbazine, prednisone; OPP, vincristine, procarbazine, prednisone; PROMACE, cyclophosphamide, doxorubicin, etoposide, prednisone, methotrexate; VAPP, vincristine, doxorubicin, procarbazine, prednisone; VIM, etoposide, ifosfamide, methotrexate.

© 2023 Geurts YM et al. *JAMA Oncol.*

**eTable 5. Patient and treatment characteristics by radiation field**			
Patient- and treatment characteristics	No subdiaphragmatic RT No. (%)	PAO (±spleen) No. (%)	Inverted Y or iliac alone (±spleen) No. (%)
All patients	171 (54.1)	55 (17.4)	90 (28.5)
Sex			
Male	107 (62.6)	37 (67.3)	77 (85.6)
Female	64 (37.4)	18 (32.7)	13 (14.4)
Period of HL diagnosis			
1964-1976	80 (46.8)	24 (43.6)	31 (34.4)
1977-1988	60 (35.1)	27 (49.1)	43 (47.8)
1989-2000	31 (18.1)	4 (7.3)	16 (17.8)
Age at HL diagnosis, years			
15-24	39 (22.8)	25 (45.5)	28 (31.1)
25-34	58 (33.9)	10 (18.2)	29 (32.2)
35-50	74 (43.3)	20 (36.4)	33 (36.7)
Follow-up time, years[a]			
5-14	26 (15.2)	10 (18.2)	17 (18.9)
15-24	52 (30.4)	18 (32.7)	29 (32.2)
25-34	64 (37.4)	19 (34.6)	38 (42.2)
≥35	29 (17.0)	8 (14.6)	6 (6.7)
RT dose to affected colon segment, Gy[b]			
No RT-<1.0	167 (97.7)	30 (54.6)	2 (2.2)
1.0-9.9	2 (1.2)	11 (20.0)	15 (16.7)
10.0-19.9	0 (0.0)	4 (7.3)	20 (22.2)
20.0-29.9	0 (0.0)	7 (12.7)	26 (28.9)
≥30.0	0 (0.0)	3 (5.5)	27 (30.0)
Unknown	2 (1.2)	0 (0.0)	0 (0.0)
Alkylating chemotherapy			
No	59 (34.5)	39 (70.9)	30 (33.3)
Yes	112 (65.5)	16 (29.1)	60 (66.7)
Anthracyclines			
No	120 (70.2)	49 (89.1)	60 (66.7)
Yes	51 (29.8)	6 (10.9)	30 (33.3)
Procarbazine dose, g/m^2 [c]			
No procarbazine	70 (40.9)	40 (72.7)	30 (33.3)
1.0-4.2	23 (13.5)	6 (10.9)	15 (16.7)
4.3-8.4	48 (28.1)	6 (10.9)	23 (25.6)
>8.4	30 (17.5)	3 (5.5)	22 (24.4)

[a] Time from HL treatment to colorectal cancer (cases) or cut-off date (controls)
[b] Matched segment for controls
[c] Assuming a procarbazine dose of 1.4 g/m^2 per cycle (14 days x 0.1 g/m^2 per day), 4.2 g/m^2 corresponds to three and 8.4 g/m^2 to six cycles of MOPP. Other protocols (e.g. MOPP/ABV) include a procarbazine dose of 0.7 g/m^2 per cycle.
ABV, doxorubicin, bleomycin, vinblastine; HL, Hodgkin lymphoma; Gy, Gray; MOPP, mechlorethamine, vincristine, procarbazine, prednisone; PAO, para-aortic; No., number; RT, radiotherapy.

© 2023 Geurts YM et al. *JAMA Oncol.*

eTable 6. Risk of CRC after HL in relation radiation dose to the whole bowel and cumulative procarbazine dose (model 1) or affected large bowel segment and cumulative procarbazine dose (model 2)					
	Treatment factor	Cases No. (%)	Controls No. (%)	Rate ratio[a] (95% CI)	P_{trend}[b]
1	RT dose to whole large bowel, Gy				<0.001
	No RT-<1.0 (median 0.22 Gy)	28 (36.4)	130 (55.6)	Ref.	
	1.0-9.9 (median 6.3 Gy)	13 (16.9)	31 (13.3)	2.1 (1.0-4.6)[ns]	
	10.0-19.9 (median 15.3 Gy)	17 (22.1)	43 (18.4)	2.6 (1.2-5.6)	
	≥20.0 (median 25.0 Gy)	19 (24.7)	30 (12.8)	3.2 (1.5-6.6)	
	Procarbazine dose, g/m^2 [c]				0.001
	No procarbazine	27 (35.1)	110 (47.0)	Ref.	
	1.0-4.2 (median 4.2 g/m^2)	9 (11.7)	34 (14.5)	1.1 (0.4-2.8)	
	4.3-8.4 (median 8.4 g/m^2)	20 (26.0)	56 (23.9)	1.9 (0.9-3.8)	
	>8.4 (median 14.0 g/m^2)	21 (27.3)	34 (14.5)	2.9 (1.4-6.1)	
2	RT dose to affected segment, Gy[d]				0.001
	No RT-<1.0 (median 0.01 Gy)	38 (49.4)	160 (68.4)	Ref.	
	1.0-9.9 (median 3.4 Gy)	7 (9.1)	19 (8.1)	1.7 (0.6-4.7)	
	10.0-19.9 (median 14.1 Gy)	8 (10.4)	16 (6.8)	2.3 (0.9-6.1)	
	20.0-29.9 (median 24.8 Gy)	12 (15.6)	21 (9.0)	2.6 (1.1-6.3)	
	≥30.0 (median 35.4 Gy)	12 (15.6)	18 (7.7)	3.2 (1.3-7.9)	
	Procarbazine dose, g/m^2 [c]				0.009
	No procarbazine	27 (35.1)	110 (47.0)	Ref.	
	1.0-4.2 (median 4.2 g/m^2)	9 (11.7)	34 (14.5)	0.9 (0.4-2.5)	
	4.3-8.4 (median 8.4 g/m^2)	20 (26.0)	56 (23.9)	1.6 (0.8-3.3)	
	>8.4 (median 14.0 g/m^2)	21 (27.3)	34 (14.5)	2.3 (1.1-4.8)	

[a] Rate ratios for development of CRC were calculated conditional on matched sets. Matching variables were sex, age at HL diagnosis and date of HL diagnosis.
[b] Test for linear trend, one dose-variable was included as continuous and the other was kept categorical. Patients with unknown radiation dose were excluded from the test for linear trend in radiation dose. If radiation dose for a case was unknown (n=1), the full case-control set was excluded from analysis
[c] Assuming a procarbazine dose of 1.4 g/m^2 per cycle (14 days x 0.1 g/m^2 per day), 4.2 g/m^2 corresponds to three cycles and 8.4 g/m^2 to six cycles of MOPP. Other protocols (e.g. MOPP/ABV) include a procarbazine dose of 0.7 g/m^2 per cycle.
[d] Mean radiation dose estimated to the affected large bowel segment (matched segment for controls)
ABV, doxorubicin, bleomycin, vinblastine; CI, confidence interval; CRC, colorectal cancer; HL, Hodgkin lymphoma; Gy, Gray; MOPP, mechlorethamine, vincristine, procarbazine, prednisone; No., number; ns, not statistically significant p>0.05; Ref, reference category; RT, radiation.

© 2023 Geurts YM et al. *JAMA Oncol.*

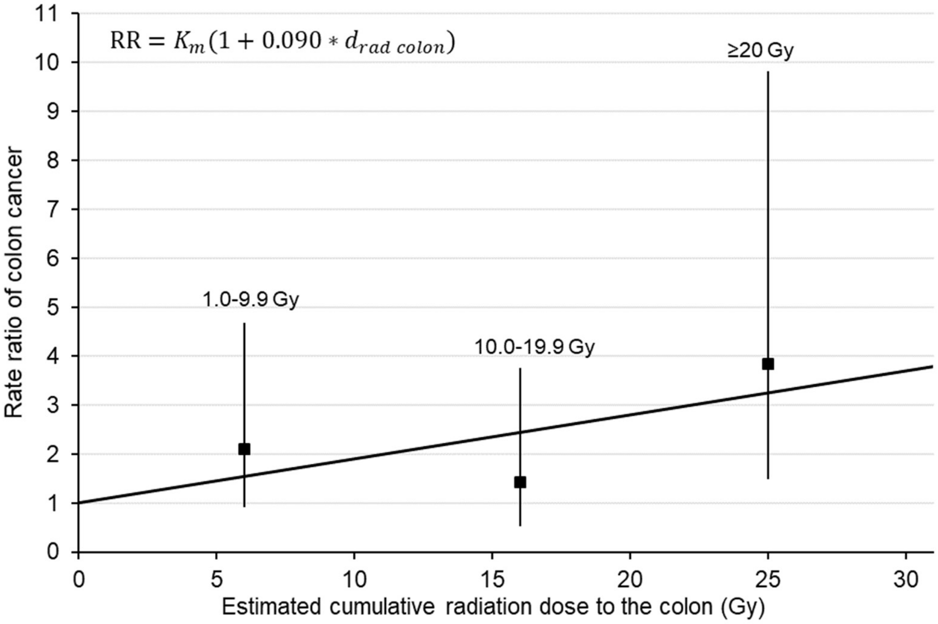

	Cases No. (%)	Controls No. (%)	Rate ratio (95% CI)	$P_{heterogeneity}$
RT dose to the colon, Gy				0.02
No RT-<1.0 (median 0.2 Gy)	21 (37.5)	95 (56.6)	Ref.	
1.0-9.9 (median 6.2 Gy)	13 (23.2)	25 (14.9)	2.1 (0.9-4.7)	
10.0-19.9 (median 16.7 Gy)	9 (16.1)	32 (19.1)	1.4 (0.5-3.7)	
≥20.0 (median 25.0 Gy)	12 (21.4)	15 (8.9)	3.8 (1.5-9.8)	
Unknown	1 (1.8)	1 (0.6)	-	

eFigure 4. The modeled overall radiation dose-response relationship for colon cancer following Hodgkin lymphoma with mean radiation dose to the colon

This model represent the average increase in colon cancer rate for radiation dose to the colon for patients included in this case-control study. The modifying effect of cumulative procarbazine dose or other variables has not been taken into account in this model. Overall, colon cancer rate increased linearly with increasing radiation dose to the colon with an ERR/Gy of 9.0% (95% CI 1.6%-26.6%). For category estimates, no radiotherapy or a mean radiation dose to the colon less than 1.0 Gy was used as reference. Filled squares and error bars indicate rate ratios and 95% confidence intervals for radiotherapy dose categories, plotted at the median dose of each category (reference category with median dose 0.2 Gy not shown).

ERR, excess rate ratio; CI, confidence interval; $d_{rad\ colon}$, radiation dose to the colon; Gy, Gray; No., number; Ref., reference category; RR, rate ratio; RT, radiation

© 2023 Geurts YM et al. *JAMA Oncol.*

**eTable 7. Overview of model specifications**				
	Model variables	Estimate (95% CI)	Formula	AIC
1	$d_{rad\ whole}$	0.092 (0.025-0.23)	$RR=K_m(1+0.092 \cdot d_{rad\ whole})$	205.0
2	$d_{rad\ whole}$<br>$d_{rad\ whole} \cdot d_{proc}$	0.034 (-0.011-0.13)<br>0.019 (0.006-0.054)	$RR=K_m(1+0.034 \cdot d_{rad\ whole}+0.019 \cdot d_{rad\ whole} \cdot d_{proc})$	196.0
3[a]	$d_{rad\ whole}$<br>d_{proc}	0.035 (0.004-0.13)<br>0.173 (0.060-0.29)	$RR=K_m(1+0.035 \cdot d_{rad\ whole} \cdot e^{0.173 d_{proc}})$	193.0
4	$d_{rad\ seg}$	0.075 (0.023-0.18)	$RR=K_m(1+0.075 \cdot d_{rad\ seg})$	203.3
5	$d_{rad\ seg}$<br>$d_{rad\ seg} \cdot d_{proc}$	0.022 (-0.011-0.099)<br>0.010 (0.002-0.026)	$RR=K_m(1+0.022 \cdot d_{rad\ seg}+0.010 \cdot d_{rad\ seg} \cdot d_{proc})$	198.5
6[b]	$d_{rad\ seg}$<br>d_{proc}	0.022 (0.002-0.086)<br>0.156 (0.025-0.29)	$RR=K_m(1+0.022 \cdot d_{rad\ seg} \cdot e^{0.156 d_{proc}})$	196.4

Every row represents one model.
[a] Best fitting model for mean radiation dose to the whole large bowel
[b] Best fitting model for mean radiation dose to the affected large bowel segment
AIC, Akaike information criterion; CI, confidence interval; $d_{rad\ seg}$, mean radiation dose to the affected large bowel segment; $d_{rad\ whole}$, mean radiation dose to the whole large bowel; d_{proc}, cumulative procarbazine dose; K_m, constant specific to each matched set; RR, rate ratio

© 2023 Geurts YM et al. *JAMA Oncol.*

eTable 8. Median dose and excess rate ratios for radiation to the whole large bowel according to patient- and treatment-related factors.								
Patient- and treatment related factors			Subdiaphragmatic RT received, no. (%)		Median mean dose to the whole large bowel, Gy (IQR)[a]		ERR per Gy, % (95% CI)[b]	$P_{heterogeneity}$
	Cases	Controls	Cases	Controls	Cases	Controls		
Overall	78 (100)	238 (100)	48 (61.5)	97 (40.8)	8.8 (0.6-20.2)	1.6 (0.3-15.6)	9.2 (2.5-23.0)	
Sex								0.1
Male	54 (69.2)	167 (70.2)	34 (63.0)	80 (47.9)	11.0 (0.8-20.2)	10.3 (0.5-17.7)	4.8 (-0.3-16.9)	
Female	24 (30.8)	71 (29.8)	14 (58.3)	17 (23.9)	3.8 (0.3-21.8)	0.3 (0.2-3.0)	24.2 (4.7-94.9)	
Age at HL diagnosis, years								>0.5
15-24	21 (26.9)	71 (29.8)	18 (85.7)	35 (49.3)	19.2 (7.6-25.7)	3.0 (0.5-17.1)	19.9 (2.7-96.3)	
25-34	24 (30.8)	73 (30.7)	12 (50.0)	27 (37.0)	3.3 (0.6-19.8)	0.8 (0.3-12.8)	5.1 (-1.2-24.6)	
35-50	33 (42.3)	94 (39.5)	18 (54.6)	35 (37.2)	6.6 (0.3-14.8)	2.3 (0.2-15.3)	8.0 (-0.4-31.9)	
Follow-up interval, years[c]								>0.5
5-14	15 (19.2)	38 (16.0)	8 (53.3)	19 (50.0)	6.0 (0.3-15.4)	7.2 (0.2-13.9)	6.3 (-2.7-59.9)	
15-24	23 (29.5)	76 (31.9)	17 (73.9)	30 (39.5)	12.9 (0.8-20.2)	0.9 (0.3-17.7)	14.1 (0.5-97.0)	
25-34	29 (37.2)	92 (38.7)	18 (62.1)	39 (42.4)	12.7 (0.6-21.0)	1.8 (0.5-17.1)	7.0 (-0.2-27.6)	
≥35	11 (14.1)	32 (13.5)	5 (45.5)	9 (28.1)	6.5 (1.2-23.8)	0.7 (0.3-12.2)	13.9 (-1.2-141.0)	
Alkylating chemotherapy								0.05
No	25 (32.0)	103 (43.3)	18 (72.0)	51 (49.5)	11.0 (1.6-20.2)	2.4 (0.5-17.6)	4.6 (-0.6-16.3)	
Yes	53 (68.0)	135 (56.7)	30 (56.6)	46 (34.1)	7.6 (0.6-20.3)	0.8 (0.2-14.9)	16.3 (5.0-42.9)	
Anthracyclines								>0.5
No	57 (73.1)	172 (72.3)	37 (64.9)	72 (41.9)	12.7 (0.8-21.8)	1.8 (0.5-17.3)	9.0 (2.3-23.1)	
Yes	21 (26.9)	66 (27.3)	11 (52.4)	25 (37.9)	6.4 (0.3-14.2)	0.4 (0.1-12.8)	10.2 (-0.2-38.9)	
Procarbazine dose, g/m^2								0.001
No procarbazine	27 (34.6)	113 (47.5)	18 (66.7)	52 (46.0)	8.8 (0.4-20.2)	1.8 (0.5-16.7)	4.1 (-0.8-15.3)	
1.0-4.2	9 (11.5)	35 (14.7)	5 (55.6)	16 (45.7)	6.0 (0.2-13.5)	1.9 (0.1-14.3)	3.7 (-3.9-27.2)	
4.3-8.4	21 (26.9)	56 (23.5)	8 (38.1)	21 (37.5)	0.9 (0.4-10.3)	8.0 (0.3-15.3)	5.4 (-1.9-28.9)	
>8.4	21 (26.9)	34 (14.3)	17 (81.0)	8 (23.5)	18.9 (9.8-22.3)	0.6 (0.3-19.7)	83.4 (22.9-317.0)	

For each patient- or tumor related factor a separate model was fitted. The ERR/Gy for patient- and treatment characteristics was modelled as:
$RR = K_m(1 + \beta_{category\ of\ characteristic} \cdot d_{rad\ whole})$

[a] Includes only patients who had radiation as part of HL treatment.

[b] Includes only patients for whom radiation dose to the large bowel was known. If radiation dose for a case was unknown, the full case-control set was excluded from analysis. Confidence intervals are based on the profile likelihood.

[c] Time from HL treatment to CRC (cases) or cut-off date (controls)

© 2023 Geurts YM et al. *JAMA Oncol.*

CI, confidence interval; $d_{rad\ whole}$, radiation dose to the whole large bowel; ERR, excess rate ratio; Gy, Gray; HL, Hodgkin lymphoma; IQR, interquartile range; no., number; RT, radiation.

**eTable 9. Median dose and excess rate ratios for radiation to the affected large bowel segment according to patient- and treatment-related factors.**								
Patient- and treatment related factors			Subdiaphragmatic RT received, no. (%)		Median mean dose to affected large bowel segment, Gy (IQR)[a]		ERR per Gy, % (95% CI)[b]	$P_{heterogeneity}$
	Cases	Controls	Cases	Controls	Cases	Controls		
Overall	78 (100)	238 (100)	48 (61.5)	97 (40.8)	3.4 (0.1-26.0)	0.4 (0.0-13.3)	7.5 (2.3-17.8)	
Sex								0.02
Male	54 (69.2)	167 (70.2)	34 (63.0)	80 (47.9)	3.4 (0.1-26.0)	0.8 (0.1-19.7)	3.5 (0.0-11.2)	
Female	24 (30.8)	71 (29.8)	14 (58.3)	17 (23.9)	8.1 (0.0-26.8)	0.2 (0.0-0.6)	39.1 (8.3-162.3)	
Sex, no pelvic RT								>0.5
Male	31 (67.4)	113 (62.8)	11 (35.5)	26 (23.0)	0.1 (0.0-0.8)	0.2 (0.0-0.6)	33.6 (-1.1-305.6)	
Female	15 (32.6)	67 (37.2)	5 (33.3)	13 (19.4)	0.1 (0.0-0.8)	0.2 (0.0-0.5)	28.3 (-3.4-225.5)	
Age at HL diagnosis, years								0.1
15-24	21 (26.9)	71 (29.8)	18 (85.7)	35 (49.3)	26.0 (13.7-30.5)	0.6 (0.2-18.9)	26.8 (5.9-111.1)	
25-34	24 (30.8)	73 (30.7)	12 (50.0)	27 (37.0)	1.9 (0.0-24.3)	0.3 (0.0-10.9)	5.5 (-0.6-24.6)	
35-50	33 (42.3)	94 (39.5)	18 (54.6)	35 (37.2)	0.2 (0.0-10.2)	0.2 (0.0-12.8)	2.1 (-1.4-12.5)	
Follow-up interval[c], years								>0.5
5-14	15 (19.2)	38 (16.0)	8 (53.3)	19 (50.0)	0.3 (0.0-26.0)	7.9 (0.0-21.4)	1.7 (-2.5-20.7)	
15-24	23 (29.5)	76 (31.9)	17 (73.9)	30 (39.5)	5.8 (0.1-19.4)	0.3 (0.0-13.8)	5.7 (-0.7-28.3)	
25-34	29 (37.2)	92 (38.7)	18 (62.1)	39 (42.4)	5.1 (0.0-31.0)	0.5 (0.1-10.7)	11.0 (1.9-40.2)	
≥35	11 (14.1)	32 (13.5)	5 (45.5)	9 (28.1)	0.8 (0.4-23.1)	0.3 (0.0-0.9)	18.0 (-0.4-217.7)	
Alkylating chemotherapy								0.2
No	25 (32.0)	103 (43.3)	18 (72.0)	51 (49.5)	3.3 (0.1-27.4)	0.4 (0.1-4.2)	4.1 (-0.4-15.2)	
Yes	53 (68.0)	135 (56.7)	30 (56.6)	46 (34.1)	6.5 (0.0-25.9)	0.3 (0.0-19.1)	9.9 (3.0-24.6)	
Anthracyclines								>0.5
No	57 (73.1)	172 (72.3)	37 (64.9)	72 (41.9)	3.4 (0.1-26.8)	0.4 (0.1-9.5)	7.7 (2.2-18.8)	
Yes	21 (26.9)	66 (27.3)	11 (52.4)	25 (37.9)	3.3 (0.0-16.7)	0.2 (0.0-14.2)	6.7 (-0.1-27.6)	
Procarbazine dose, g/m^2								0.009
No procarbazine	27 (34.6)	113 (47.5)	18 (66.7)	52 (46.0)	0.8 (0.1-27.4)	0.4 (0.1-3.0)	3.6 (-0.7-14.2)	
1.0-4.2	9 (11.5)	35 (14.7)	5 (55.6)	16 (45.7)	0.2 (0.0-13.9)	0.2 (0.0-13.8)	1.9 (-2.1-18.0)	
4.3-8.4	21 (26.9)	56 (23.5)	8 (38.1)	21 (37.5)	0.1 (0.0-20.8)	0.4 (0.0-21.2)	3.2 (-1.4-16.1)	
>8.4	21 (26.9)	34 (14.3)	17 (81.0)	8 (23.5)	21.8 (5.4-29.3)	0.5 (0.0-18.6)	50.2 (13.2-211.4)	

© 2023 Geurts YM et al. *JAMA Oncol.*

For each patient- or tumor related factor a separate model was fitted. The ERR/Gy for patient- and treatment characteristics was modelled as:

$RR=K_m(1+\beta_{category\ of\ characteristic} \cdot d_{rad\ seg})$

[a] Includes only patients who had radiation as part of HL treatment.

[b] Includes only patients for whom radiation dose to the affected large bowel segment (matched segment for controls) was known. If radiation dose for a case was unknown, the full case-control set was excluded from analysis. Confidence intervals are based on the profile likelihood.

[c] Time from HL treatment to CRC (cases) or cut-off date (controls)

CI, confidence interval; $d_{rad\ seg}$, radiation dose to the affected large bowel segment; ERR, excess rate ratio; Gy, Gray; HL, Hodgkin lymphoma; IQR, interquartile range; K_m, constant specific to each matched set; no., number; RT, radiation.

© 2023 Geurts YM et al. *JAMA Oncol.*
