# Supplementary material for: Association of Radiation and Procarbazine Dose With Risk of Colorectal Cancer Among Survivors of Hodgkin Lymphoma
Source: JAMA Oncol. 2023 Feb 2;9(4):481–9. doi: 10.1001/jamaoncol.2022.7153 (PMC9896374; doi:10.1001/jamaoncol.2022.7153)
Supplement: Supplement 2. — Data Sharing Statement [file jamaoncol-e227153-s002.pdf]

Data Sharing Statement

Geurts. Association of Radiation and Procarbazine Dose With Risk of Colorectal Cancer Among Survivors of Hodgkin Lymphoma. *JAMA Oncol*. Published February 02, 2023. doi:10.1001/jamaoncol.2022.7153

Data
Data available: No
